# Genetic variability in the *sdrD* gene in *Staphylococcus aureus* from healthy nasal carriers

**DOI:** 10.1186/s12866-018-1179-7

**Published:** 2018-04-16

**Authors:** Clement Ajayi, Espen Åberg, Fatemeh Askarian, Johanna U. E. Sollid, Mona Johannessen, Anne-Merethe Hanssen

**Affiliations:** Research group of Host-Microbe Interactions, Department of Medical Biology, Faculty of Health Sciences, UiT-The Arctic University of Norway, 9037 Tromsø, Norway

**Keywords:** *S. aureus*, Sdr, Healthy carrier, Virulence factors, Allelic variants

## Abstract

**Background:**

*Staphylococcus aureus* cell wall anchored Serine Aspartate repeat containing protein D (SdrD) is a member of the microbial surface component recognising adhesive matrix molecules (MSCRAMMs). It is involved in the bacterial adhesion and virulence. However the extent of genetic variation in *S. aureus sdrD* gene within isolates from healthy carriers are not known. The aim of this study was to evaluate allelic variation of the *sdrD* gene among *S. aureus* from healthy nasal carriers.

**Results:**

The *sdrD* A region from 48 *S. aureus* isolates from healthy carriers were analysed and classified into seven variants. Variations in the *sdrD* A region were concentrated in the N2 and N3 subdomains. Sequence analysis of the entire *sdrD* gene of representative isolates revealed variations in the SD repeat and the EF motifs of the B repeat. In silico structural modelling indicates that there are no differences in the SdrD structure of the 7 variants. Variable amino acid residues mapped onto the 3D structure revealed that the variations are surface located, exist within the groove between the N2-N3 subdomains and distributed mainly on the N3 subdomain. Comparison of adhesion to keratinocytes in an in vitro cell adhesion assay, using NCTC 8325–4*∆sdrD* strains expressing the various *sdrD* gene variants, indicated a significant difference between only two complements while others showed no major difference in their adhesion.

**Conclusions:**

This study provides evidence of sequence variations across the different domains of SdrD from *S. aureus* isolated from healthy nasal carriers. Proper understanding of these variations is necessary in the study of *S. aureus* pathogenesis.

**Electronic supplementary material:**

The online version of this article (10.1186/s12866-018-1179-7) contains supplementary material, which is available to authorized users.

## Background

*Staphylococcus aureus* is an opportunistic human microbe, often found in the anterior nares of one out of four healthy adult individuals [1, 2]. It is responsible for a wide range of human diseases including skin infections such as folliculitis, impetigo and more severe diseases such as endocarditis, and septicaemia [3]. Despite antibiotics use and improved health care, *S. aureus* still remains one of the major causes of hospital-related infections. [4]

**Fig. 1:**
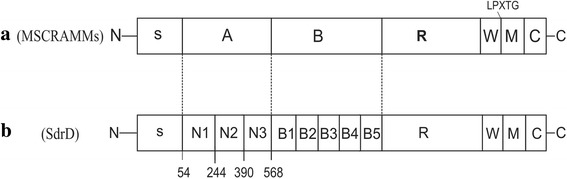
Schematic representation of MSCRAMMS and the SdrD of *S. aureus* NCTC8325 (**a**) MSCRAMMs have a common structural domain organisation consisting of a signal sequence (S) followed by a ligand binding domain (A), B repeat region (B), Serine-aspartic acid repeat region (R), wall-spanning region (W), LPXTG motif; transmembrane region (M); cytoplasmic domain (C). (**b**) Representation of full length SdrD protein from *S. aureus* NCTC8325. Details of the SdrD A subdomains N1 (54–243), N2 (244–390) and N3 (319–568) are indicated. The B repeat region consists of five repeats (B1-B5)

Critical in *S. aureus* pathogenesis is its adherence to host cells and/or components present in the host’s extracellular matrix [5]. *S. aureus* expresses an array of virulence factors such as the Microbial Surface Component Recognising Adhesive Matrix Molecules (MSCRAMMs), which facilitate its successful adherence [6]. These proteins include surface proteins such as clumping factor (Clf) A, ClfB, serine-aspartate repeat containing protein C (SdrC), SdrD and SdrE. They share similar structural organisation consisting of an N-terminal secretory signal peptide, followed by an A domain, B repeat, and R region containing serine-aspartate repeats. The C- terminal consists of an LPXTG cell wall-anchoring motif, hydrophobic membrane-spanning region and a charged cytoplasmic tail [6] (Fig. 1). These proteins interact with host molecules such as fibrinogen [7], desmoglein 1 (Dsg 1) [8] and β-neurexin [9]. Overall, they contribute to *S. aureus* virulence, immune evasion and survival within the host [6].

*S. aureus* SdrD is encoded within the *sdr* locus in a tandem arrangement with *sdrC* and/or *sdrE* genes [10]. A previous study indicated that while *sdrC* was almost always present in all the *S. aureus* strains studied, *sdrD* had a prevalence of 60% [11]. The different regions within the SdrD protein structure (Fig. 1) contribute to its overall function. The signal peptide directs its translocation to specific sites within the peptidoglycan of the bacterial cell wall [12] The A region is considered to be the ligand binding region and is further divided into three subdomains N1, N2 and N3 [6, 13]. The N2 and N3 subdomains bind the ligand via a “dock-lock-latch” mechanism [13]. In this process, the ligand docks within the groove between these domains, the N3 subdomain locks the ligand in place and provides an additional structure that latches onto the N2 subdomain, further locking the ligand in place. The SdrD B repeat is made up of B1-B5 subdomains of 110–113 amino acid residues, which contains an EF motif that interacts with calcium ions [14]. It is proposed that the B repeat functions as a spacer influencing the distance between the ligand binding A region and the bacterial cell surface [15].

Several studies have delineated the function and expression profile of the SdrD protein, further emphasising its importance in *S. aureus* virulence. It is expressed during nasal colonisation [16] and increase bacterial adherence to desquamated nasal cells [17]. Moreover, SdrD promotes the adhesion of *S. aureus* to keratinocytes via its interaction with Dsg1 [8]. *sdrD* expression is increased in the presence of blood [18] and it was recently shown that the protein also promotes bacterial survival and virulence during systemic infection [19]. Moreover, a correlation between the presence of the *sdrD* gene and bone infections has been observed [11, 20].

Genetic variation within genes encoding virulence factors may influence bacterial pathogenesis. Polymorphisms in the A domain of FnBP A and B in *S. aureus* have been reported [21, 22] and specific single amino acid polymorphisms in FnBP A is associated with infection of cardiovascular devices [23]. Xue et al. [24] observed polymorphisms in the *sdrD* gene from clinical or sub-clinical bovine mastitis associated *S. aureus* isolates. Furthermore, genetic variations within *S. aureus sdrD* sequences obtained from GenBank have also been reported [25]. Sources of the *S. aureus* isolates used in the aforementioned studies were either from animal or diseased humans. However, genetic variability in the *sdrD* gene in *S. aureus* isolates from a healthy human population has not been reported. The aim of this study was to determine sequence variability in the *sdrD* gene among *S. aureus* isolated from anterior nares of healthy adult carriers. Furthermore, we aimed to further characterise these variations within the structural domains of the SdrD proteins and evaluate the possible effects of these variations on bacterial adhesion.

## Results

### Prevalence of genes encoding *sdrC, sdrD* and *sdrE* in *S. aureus*

The prevalence of the three *sdr* genes in 554 *S. aureus* isolates is shown in Table 1. The *sdrC* gene was present in 99% of the isolates investigated while *sdrD* and *sdrE* genes were present in 29% and 84% of the isolates, respectively. Furthermore, the combination of the genes varied across the isolates. Among the 554 isolates, four isolates were negative for the three genes, 146 isolates carried all three, 328 isolates carried two and 76 isolates carried only one. None of the isolates carried the *sdrD* gene as a single gene.

**Table 1:** Prevalence of *sdr* genes within *S. aureus* isolates from healthy carriers (*n* = 554)

Gene	*sdrC*	*sdrD*	*sdrE*	Number of isolates (n)
Gene combination	+	+	+	146
	+	+	–	11
	+	–	+	316
	–	+	+	1
	+	–	–	74
	–	+	–	0
	–	–	+	2
	–	–	–	4
Total Number of positive isolates n (%)	547 (99%)	158 (29%)	465 (84%)	554

The first 51 *sdrD* positive isolates were selected for further analysis. The selected isolates were distributed into eight *spa* clonal complexes (*spa* CC) (CC2, CC5, CC24, CC65, CC78, CC84, CC267, CC153) (Table 2). In addition, there were two singletons that could not be clustered. The number of isolates within each *spa* CC varied but the majority of the isolates were found in CC84.

**Table 2:** Characterization of the *sdrD* positive *S. aureus* strains isolated from anterior nares of healthy human analyzed in this study

*spa*-Clonal Complexes (CC)	Number of isolates	Strain ID no	*spa* type*
CC2	7	15,19	t002
		14	t045
		26	t5423
		51	t548
		17	t581
		12	t601
CC5	4	46,16	t005
		3	t223
		44	t309
CC24	7	20,25	t008
		35,36	t024
		13	t1476
		4	t305
CC65	1	43	t209
CC78	10	42	t078
		10,22	t1102
		6	t167
		39	t280
		7	t349
		27	t5257
		2	t5449
		5	t759
		30	t814
CC84	16	1,11,23,28,29,32,33,34,37,41,46,48,50	t084
		38	t094
		31	t2219
		18	t5232
CC153	1	40	t153
CC267	1	24	t4173
Singleton	2	9	t3160
		49	t186
N/A	2	21	t258
		45	N/A

* Results from *spa* typing are from a previous study [36]

### Sequence alignment and phylogenetic analyses of the *sdrD* A-domain

To investigate the sequence diversity of the *sdrD* gene, a region of approximately 1500 bp covering the *sdrD* A region was sequenced from the 51 *sdrD* positive *S. aureus* isolates. Three of the isolates did not yield good sequences and therefore they were excluded from further analysis. Nucleotide sequences and the predicted amino acid sequences from the remaining 48 *S. aureus* isolates were aligned with the corresponding sequences from six *S. aureus* genomic sequences (NCTC8325, N3I5, MW2, MSSA476, Newman and HO 5096 0412).

The alignment revealed a considerable diversity across the sequences with variations in form of nucleotide transitions, transversions, deletions and insertions (Additional file 1). However, similarities were also found. The *sdrD* A region in isolates 12, 14 and 15 were identical to that in N315, while the *sdrD* A-region in isolates 13 and 36 showed 100% identity with *sdrD* in NCTC8325.

**Fig. 2:**
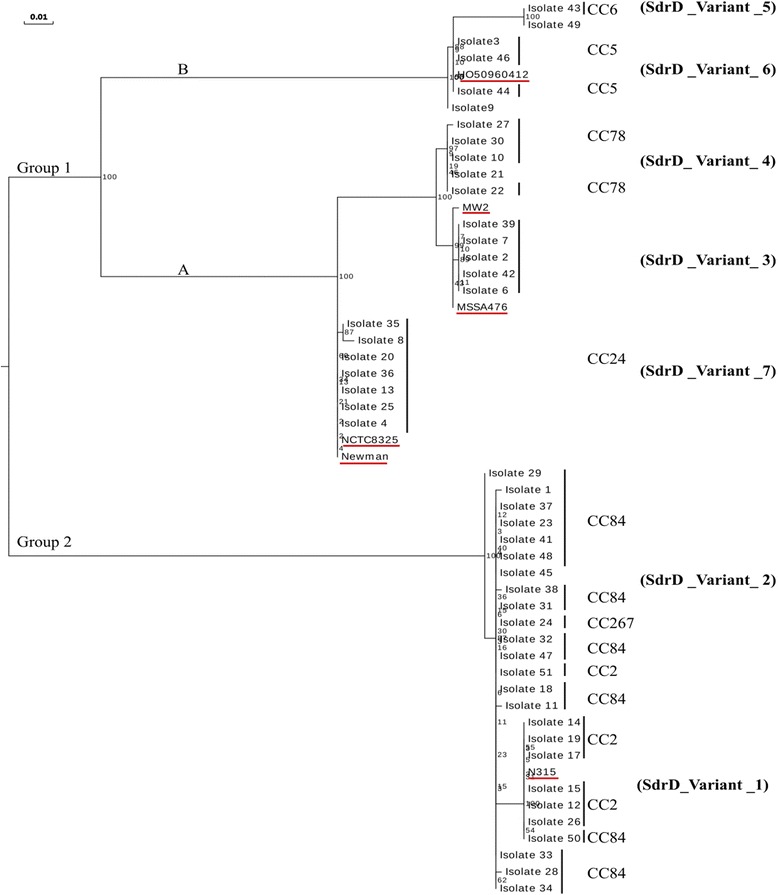
Phylogenetic analysis of the *sdrD* A region in *S. aureus*. The region encoding the *sdrD* A domain was sequenced in 48 *S.aureus* isolates from healthy individuals. Six *S. aureus* reference sequences (underlined in red) were also included. Phylogenetic relatedness was inferred from the predicted amino acid sequences by the maximum likelihood method using RaxML [27]. Bootstrap support values (1000 replicates) are indicated on the nodes. *Spa* clonal complexes (*spa* CCs) and *sdrD* variants are also indicated

Figure 2 shows a maximum likelihood tree based on the deduced amino acid sequences of the *sdrD* A region of the 48 queried and 6 reference strains. The phylogenetic tree split into two major groups, indicated as group 1 and 2. Group 1 further separated into two sub-groups, indicated as A and B, these further divided into smaller clusters. Based on the tree topology, isolates 43 and 49 showed the highest divergence for group 1. The reference sequences included in our analysis were distributed across all clusters. Furthermore, the strains also clustered according to their *spa* clonal complexes. The phylogenetic analysis based on the *sdrD* nucleotide sequences resulted in a similar pattern of clustering and divergence (data not shown).

Based on the observed diversity in the A domain of the *sdrD* gene, we classified the isolates into different variants (Fig. 2). For each *sdrD* variant, a representative isolate was randomly selected. *S. aureus* NCTC8325–4 was selected as the representative strain of the sdrD variant 7. Amino acid identity within this region between the seven *sdrD* variants was between 85% and 99% (Additional file 4: Table S1). These results indicate allelic sequence diversity in the A region of the *sdrD* gene within these *S. aureus* isolates.

### Sequence variation within the entire *sdrD* gene

Whole genome sequencing was performed on the representative isolate of each *sdrD* variant, including *S. aureus* NCTC8325–4 as it had been chosen as representative strain of variant 7. Sequences of the *sdrD* gene were extracted from all the seven and compared. A pairwise comparison of the deduced amino acid sequences showed identity ranging from 93.1% - 99.3%, where *sdrD* variants 3, 4 and 7 were most similar to each other. The size of the SdrD polypeptides ranged from 1315 to 1399 amino acids (Table 3). We found amino acid variations in the consensus sequence of the EF motifs responsible for calcium binding [14]. For instance, in s*drD* variant 6, there were changes in amino acids residues at position 585 (Val to Iso), 591 (Val to Lys), and 594 (Gly to Lys). There were also extensive insertions or deletions within the R-domain, consisting of serine aspartate repeats. Finally, the N2-N3 domain of the A-region varied extensively, which are in agreement with results shown in Fig. 2. In contrast, the N-terminal signal peptide, YSIRK/GS motif, the signal peptide cleavage sites [12, 26], the B repeats and the residues of the hydrophobic tail were highly conserved among the seven SdrD variants (Additional file 2).

**Table 3:** Percentage Amino Acid Identity using Amino acid for the entire *sdrD* gene for the seven representative *sdrD* variants

	(aa)	SdrD Variant 1	SdrD Variant 2	SdrD Variant 3	SdrD Variant 4	SdrD Variant 5	SdrD Variant 6	SdrD Variant 7
SdrD Variant 1	1365	100	99.3	93.9	93.7	94.5	93.1	94.5
SdrD Variant 2	1399	99.3	100	94.4	93.9	94.8	93.5	94.8
SdrD Variant 3	1329	93.9	94.4	100	98.7	95.3	94.5	98.5
SdrD Variant 4	1365	93.7	93.9	98.7	100	94.7	93.9	98.2
SdrD Variant 5	1315	94.5	94.8	95.3	94.7	100	97.6	95.6
SdrD Variant 6	1375	93.1	93.5	94.5	93.9	97.6	100	94.9
SdrD Variant 7	1380	94.5	94.8	98.5	98.2	95.6	94.9	100.0

**Fig. 3:**
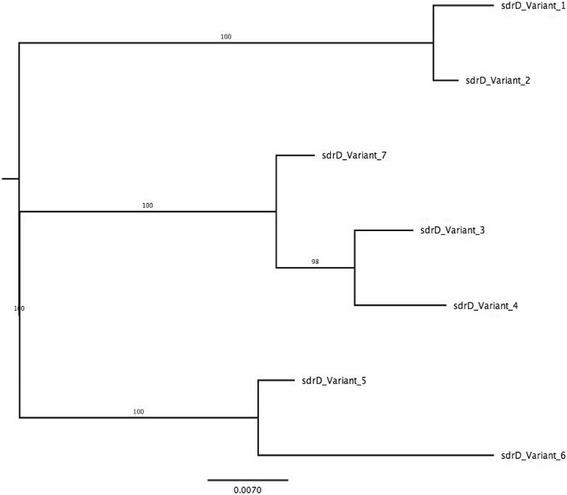
Phylogenetic relationship between the *sdrD* variants. Phylogenetic tree based on the deduced amino acid sequences for the entire *sdrD* gene from seven representative *S. aureus* strains. The tree was generated by the maximum likelihood method using RaxML [27]. The bootstrap support values (1000 replicates) are indicated on the branches. Branch length is proportional to the number of substitutions as indicated by the scale bar

Phylogenetic analysis of the deduced amino acid sequences for the entire *sdrD* gene based on the maximum likelihood method also indicated *sdrD* sequence diversity in the representative isolates (Fig. 3). The phylogenetic tree divided into three major clades. These results further affirm the genetic variability within the *sdrD* gene.

### Multilocus sequence type (MLST) analysis of *sdrD* variants

**Fig. 4:**
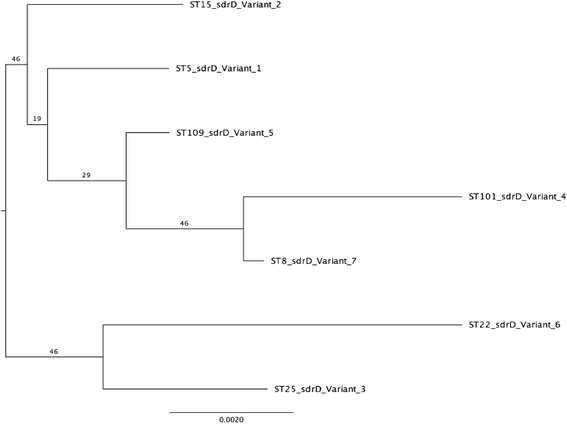
Maximum likelihood tree based on the concatenated MLST alleles. Sequences types based on the allelic profiles of the MLST alleles in seven selected *S. aureus* strains are indicated in the tree. Bootstrap support values (1000 replicates) are indicated next to the branches. Branches length is proportional to the number of substitutions as indicated by the scale bar. The *sdrD* variants are also indicated

We determined the MLST of the representative *S. aureus* isolate for each *sdrD* variant and performed a phylogenetic analysis based on this. Figure 4 shows a maximum likelihood tree based on the concatenated sequences of the seven housekeeping genes for these isolates. The isolates were distributed into seven different sequence types (ST) and the phylogeny tree split into two groups (Fig. 4). The variants were distributed randomly within the groups. The MLST phylogeny had a different topology compared to the *sdrD* phylogeny shown in Fig. 3. For instance, in the MLST tree, *S. aureus sdrD* variants 2 (ST15) and 5 (ST109) formed a clade, but clustered into different groups in the *sdrD* phylogeny. In addition, *sdrD* variants 6 and 5 were more closely related in the *sdrD* phylogeny but clustered differently in the MLST tree.

### 3D structural modelling and characterization of SdrD variants

**Fig. 5:**
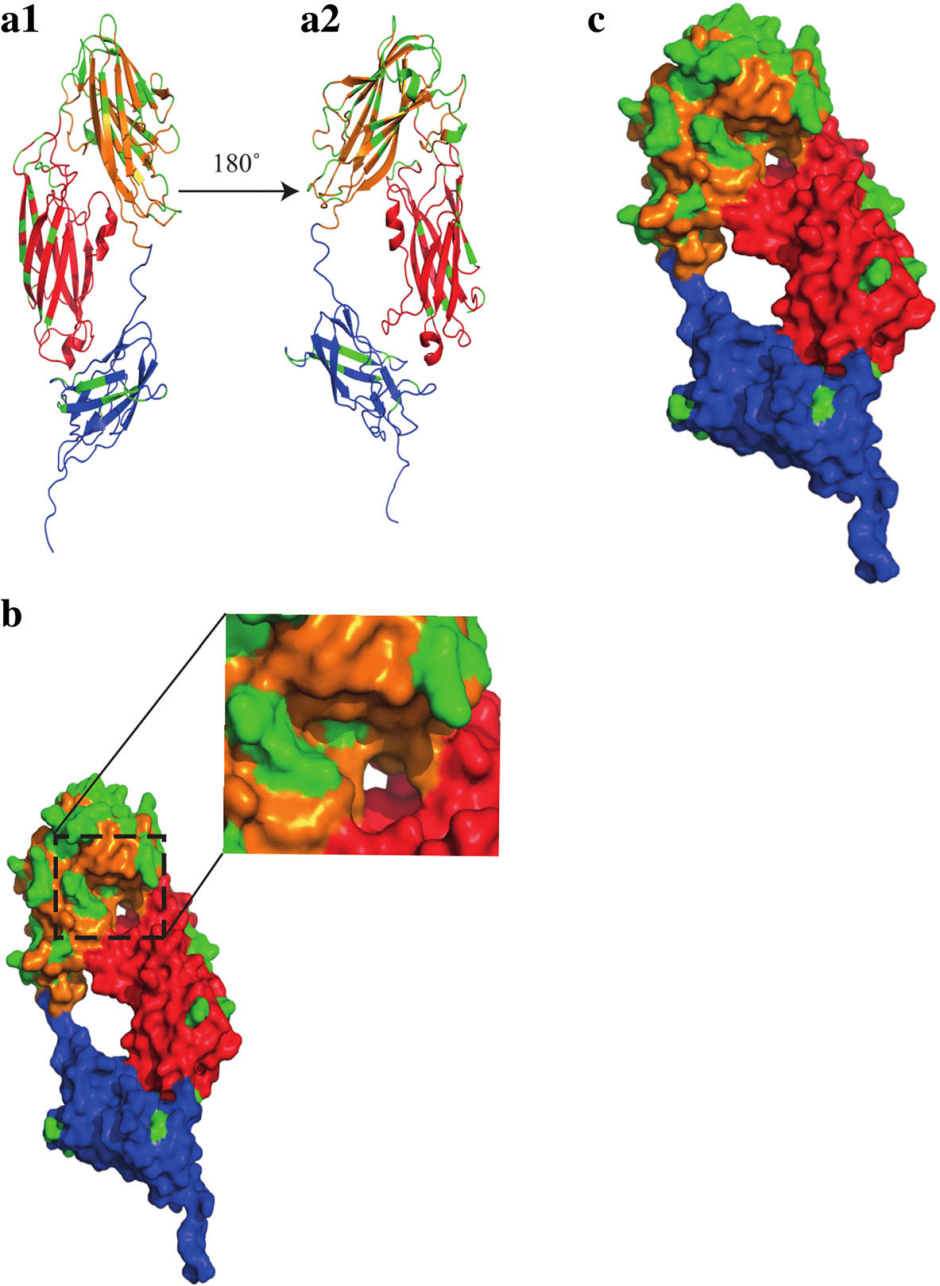
Structural characterization of the *sdrD* variants. 3D models were generated based on the N2-N3 and B1 subdomains of SdrD [15] in seven representative *S. aureus* strains. The subdomains are shown as N2 (red), N3 (orange) and B1 (Blue). Green colour coding indicates the variable amino acid residues within the seven *sdrD* variants (**a**) AI is ribbon diagram of N2-N3-B1 while A2 shows the diagram when reoriented 180 degrees. (**b**) Space filling diagram of N2-N3-B1. The grove between N2 and N3 is enlarged. (**c**) Space filling diagram of N2-N3-B1. The model shows the location of varied amino acids residues on the surface

Based on the suggested crystal structure of the N2-N3-B1 subdomains of *S. aureus* SdrD [13], 3D models of N2-N3-B1 subdomains for the seven SdrD variants were generated using the Swiss model server. Comparisons of the generated 3D structure indicated a very high structural similarity with RMSD root mean square deviation (RMSD) values ranging from 0.008–0.059 Å (data not shown). Most of the amino acid variations were located on the N3 subdomain (Fig. 5a). In addition, some of the varied amino acid residues were located within and around the groove between the N2-N3 subdomains (Fig. 5b). Furthermore, the majority of the amino acid variations were surface associated (Fig. 5c).

### Adhesion of SdrD variants to keratinocytes

SdrD have previously been shown to promote *S. aureus* adhesion to human keratinocytes [8], and we next wanted to evaluate whether the allelic variation in SdrD could influence this. In order to have identical genetic background, the *sdrD* gene variants were cloned into pMG36e. However, we encountered technical challenges in cloning the *sdrD* gene for *sdrD* variant 1, therefore we could not investigate the effect of this variant on adhesion to keratinocytes. The cloned plasmid was used to complement NCTC8325*ΔsdrD* and all had similar growth rate (Additional file 3a). The plasmids were confirmed to contain *sdrD* by direct sequencing. Complemented NCTC8325*ΔsdrD* that were confirmed to express SdrD (Additional file 3b) were used for adhesion to mammalian cells. However, we could not detect SdrD expression in variants 3 and 4, though direct sequencing and PCR on plasmid isolated from the complements, indicated the presence of the *sdrD* sequence (results not shown). Therefore, we continued our assay with *sdrD* variants 2, 5,6 and 7.

**Fig. 6:**
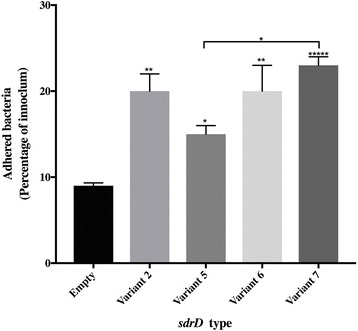
Adhesion of complemented *S. aureus* mutants to HaCaT cells. Adherence of *S. aureus* isogenic mutant NCTC8325–4*ΔsdrD* complemented with different pMG36e-SdrD variants or pMG36e (empty vector). Adherence was calculated as a percentage of the mean colony-forming unit (cfu/ml) of attached bacteria to the mean cfu/ml of the inoculum. The data represent mean of 3 independent experiments, each with 3 technical repeats. Statistical analysis was performed using one-way ANOVA with Tukey’s multiple comparison test (*P < 0.05, **P < 0.01, ***P < 0.001, ****P < 0.0001)

*S. aureus ΔsdrD* complemented with *sdrD* variants were co-incubated with HaCaT cells for 90 mins. After washing, the adhered bacteria were harvested and quantified via serial diluting and subsequent plating on blood agar. All the tested complemented *S. aureus* strains showed significant adhesion to HaCaT cells compared to the control strain lacking *sdrD* (Fig. 6). In addition, we also observed a significant difference in the adhesion of variant 5 compared to variant 7.

## Discussion

*S. aureus* is an opportunistic microbe which frequently colonizes humans using its array of virulence factors. We investigated the prevalence of the *sdr* genes among *S. aureus* isolates obtained from anterior nares of a healthy adult population. Most importantly, we report genetic variability in the *sdrD* gene in *S. aureus* isolates from healthy adults.

Sequence diversity within virulence genes in *S. aureus* have been reported; genes such as *coa* [25], *spa* [25], *agr* [27], and *fnb* [21] were reported to be polymorphic in *S. aureus* isolates. Moreover, variation in sequences encoding surface proteins expressed by *S. aureus*, are thought to be essential in its survival to environmental changes [28]. The genetic variability within the *sdrD* gene sequences varied across its defined structural domains. While the N2-N3 sub domain of the A-domain, B1 sub-domain of its B-repeats and SD repeats were highly diverse, the other regions showed a high degree of conservation. The findings are in agreement with previous studies, which report variations in the *sdrD* gene in *S. aureus* isolates from livestock [24] and sequences obtained from the GenBank database [25].

Analysis of the entire *sdrD* gene from whole genome sequencing of seven selected representative isolates also indicated regions of insertion and deletion within the R-domain, a region made of tandem repeats of serine- aspartate (SD) dipeptide [10]. The SD residues of *S. aureus* become glycosylated resulting in resistance to proteolytic cleavage in blood [29] and in pathogenesis of *S. aureus* bloodstream infections [30]. It is suggested that glycosylation intensity is dependent on the number of SD repeats available [29]. Thus, insertion of additional residues within SD repeats might have an influence on *S. aureus* pathogenicity. However, glycosylation of the repeats also primes the antibodies of the immune system in recognising these proteins [29]. Therefore, it is reasonable that SD residue deletions might be a mechanism employed by *S. aureus* to avoid detection by its host immune surveillance. A previous study has demonstrated that the SD-repeats are hot spot regions with great instability and genetic variation, which can be the result of replication slippage [31]. The genetic variability within this region might suggest the adaptation of *S. aureus* to its environment without losing the functionality of other regions within its surface proteins.

In SdrD, the YSIRK/GS motifs located at the N-terminal signal peptide direct the secretion of SdrD precursor proteins and ensure the ring-like distribution across the *S. aureus* cell wall [12, 32]. In addition, effective anchoring of the SdrD protein by the sortase A to the cell wall is ensured by the LPXTG motif [32]. These consensus sequences were highly conserved among the variants in our study, suggesting a selective pressure that drives the sequence diversity against the functional protein and not the precursor protein. Overall, the B repeat was considerably conserved among the *sdrD* variants. However, there was genetic variability within the sequences of some of the EF motifs. The conserved EF motifs of the SdrD B repeat are involved in calcium binding [14]. Calcium binding to the B repeat has been shown to be important in maintaining its structure [15] while projecting the ligand binding A domain from the bacterial surface [6, 13].

SdrD contributes in *S. aureus* adhesion to human keratinocytes [8] and to desquamated nasal epithelial cells [17]. In concordance with this, we observed that complemented *sdrD* mutants expressing the different SdrD variants exhibited significant adherence to HaCaT cells compared to the isogenic mutant harboring the empty plasmid. The adhesion of the SdrD variants was comparable to each other, except from a significant difference between variants 5 and 7. This could be explained by high diversity in the N2-N3 of the A-region and the B1 subdomain of SdrD. Analysis of these subdomains in the *sdrD* variants showed that there are sequence variations in the groove between the N2 and N3 subdomains and the variations are mostly associated with the N3 subdomain. The groove between the N2-N3 sub domains of the A-domain together with the B1 subdomain is involved in ligand interaction in a dock, latch and lock mechanism [6, 13]. It is possible that the amino acid changes within these regions have resulted in the varied adhesion abilities of the *sdrD* variants to HaCaT cells.

It is tempting to speculate what could account for the sequence variations within the different commensal *S. aureus* isolates. Our MLST phylogeny analysis revealed that *S. aureus* isolates belonging to different clades encoded considerable similar *sdrD* genes. Horizontal gene transfer (HGT), specifically homologous recombination, is suggested to underline the transfer of genes across different *S. aureus* isolates [33]. *S. aureus* virulence genes are found on Mobile genetic elements (MGE) and the potential of transfer of these elements between *S. aureus* strains are high [34]. In addition, there have been reports of transfer of non- MGE sequences between *S. aureus* strains [34]. Furthermore, polymorphism in sequences of the genes encoding FnBP A and B have been reported to arise through HGT [35].

## Conclusion

In summary, our findings reveal the genetic variability within the *sdrD* gene in *S. aureus* isolates from healthy individuals. Most importantly, our study provides an insight into how the sequences of various domains within the *S. aureus* SdrD protein are conserved. It also indicates the effect the variation might have on bacterial adhesion. However, how this might affect other reported functions of SdrD remains to be investigated.

## Materials and methods

### Bacterial strains

A total of 4000 nasal culture swabs were taken from the anterior nares of healthy adult participants recruited between 2007 and 2008 in the 6th Tromsø Study. The nasal culture swabs were taken for the purpose of research during the Tromsø Staph and Skin Study, as part of 6th Tromsø Study. Of these, 1113 isolates were confirmed as *S. aureus* and were *spa*-typed previously [36].

From the 1113 *S. aureus* isolates, we selected 554 consecutive isolates and screened for the presence of *sdrC*, *sdrD* and *sdrE* genes by PCR using primers listed in Table 4. The first 51 *sdrD* positive isolates were selected for further analysis, independent of *spa*-types or distribution of *sdr* genes. In addition *S. aureus* NCTC8325–4 and NCTC8325–4∆*sdrD* [8], was also used in the study.

**Table 4:** List of Primers used in this study

Primers	Sequence (5′ - 3′)*	Reference
Prevalence of *sdr* gene		
SdrC prevF	AAAAGGCATGATACCAAATCGA	Sabat et al. [41]
SdrC prev R	AATTCTCCATTCGTATGTTCTG	Sabat et al. [41]
SdrD prev F	AGTGGGAACAGCATCAATTTTA	Sabat et al. [41]
SdrD prev R	GTGGTAGATTGTACACTTTCT	Sabat et al. [41]
SdrE prev F	AGAAAGTATACTGTAGGAACTG	Sabat et al. [41]
SdrE prev R	GATGGTTTTGTAGTTACATCGT	Sabat et al. [41]
Sequencing of SdrD A region		
sdrD A- F	GGAACCAAGAAGCAAAGGCTG	Xue et al. [6]
sdrD A-R	CTTCTTGACCAGCTCCGCCAC	Xue et al. [6]
sdrD Fwd	AGTTGATGACAAAGTTAAATCAGGT	This study
sdrD Rwd	TAATATCTTCCGGATTCAATCCA	This study
Cloning of *sdrD*		
Sacl-amh 100 Fwd	AAAAGAGCTCTGAATTAGGAGTAATCTAATGCT	This study
Sacl-amh 101 Fwd	AAAAGAGCTCATTTGATGAATTAGGAGTAAT	This study
Sphl- amh200R	TTCAGCATGCCGCCTCATATAAGTTTTATTCCGT	This study
Sphl- amh201R	ATCAGCATGCAAATTTTGAAATAAAAAACCAGGCC	This study

*restriction sites underlined

### DNA extraction for PCR

Genomic DNA was extracted from *S aureus* using a boiling method as previously described [37], and stored at − 20 °C until use.

## PCR amplification and sequencing

The *sdrC*, *sdrD* or *sdrE* genes in the selected 554 *S. aureus* isolates were amplified by PCR using ReddyMix (Thermo Scientific, USA) according to the manufacturer’s instruction. Primers used are listed in Table 4. The cycling conditions for these were: 94 °C for 5 mins; 30 cycles of 94 °C for 10 s, 53 °C for 30 s and 72 °C for 15 s; and 72 °C for 10 mins and thereafter kept at 4 °C. The reference strain *S. aureus* N315 (NC_002745.2) was used as positive control for the amplification of these genes while *Enterococcus faecium* BM4105-RF was used as negative control.

PCR amplification of the *sdrD* A region was also carried out with ReddyMix (Thermo Scientific, USA) according to the manufacturer’s instruction. Due to limitation of our DNA sequencer to 1000 bp, amplification of approximately 1500 bp encoding the A domain was performed in two parts using the primer combinations SdrD A-F + SdrD-Rwd and SdrD-Fwd + SdrD-A-R (Table 4) resulting in PCR products of 765 bp and 840 bp, respectively. The PCR products were purified using ExoSAP-IT (Thermo Fisher Scientific, USA) according to the manufacturer’s instruction and used as template for direct sequencing using SdrD-A-F and SdrD- A-R primers (Table 4). This was performed using Big Dye Terminator 3.1 cycle sequencing kit (Applied Biosystem, USA) according to the manufacturer’s instructions.

### Sequence analysis and phylogenetic tree construction

Sequences encoding the *sdrD* A region obtained by PCR described above and *sdrD* A region sequences of six published *S. aureus* genomes obtained from the NCBI GenBank (MW2 (NC_003923.1), Newman (NC_009641.1), N315 (NC_002745.2), NCTC8325 (NC_007795.1), MSSA476 (BX571857.1) and HO 5096 0412 (NC_017763.1) were analysed further. Sequence alignment of the DNA sequences and its predicted amino sequences was performed in Bio Edit sequence alignment editor (version 7.2.5) [38] using the Cluster W algorithm program [39]. Subsequent multiple alignments were also performed using Fast Fourier Transform (MAFFT) (Version 7) [40]. Pairwise sequence alignment of predicted amino acid sequences to calculate amino acid identity was performed using the ExPASy [41].

Phylogenetic trees of the *sdrD* A domain nucleotide sequences and the deduced amino acid sequences were generated using Randomized Axelerated Maximum Likelihood (RaxML) [42]. Bootstrap values were calculated using 1000 replicates to generate confidence in the tree topology. The tree generated was viewed and mid point rooted using Fig tree (version 1.4.3) [43] and Evolview v2 [44].

### Whole genome sequencing of *S. aureus*

Genomic DNA from seven representative *S. aureus* isolates was extracted using the GenElute Bacterial Genomic DNA kit (Sigma-Aldrich, US) according to the manufacturers instruction. Samples were submitted to the Norwegian Sequencing Centre, Oslo (www.sequencing.uio.no) for Illumina HiSeq paired-end sequencing. We assessed the quality of the reads using FastQC [45]. The sequence reads were assembled using SPAdes (version 3.6.1) [46] with the --careful option. All genome assemblies were evaluated using the QUAST tool [47]. Protein coding sequences (CDSs) were predicted using MetaGeneMark [48]. The *sdr*-gene sequences were manually isolated using ARTEMIS [49].

### Multilocus sequence typing (MLST)

MLST analysis was carried out for the representative *S. aureus* isolates. Nucleotide sequences of the following house keeping genes: *arcC* (carbamate kinase), *aroE* (shikimate dehydrogenase), *glpF* (glycerol kinase), *gmk* (guanylate kinase), *pta* (phosphate acetyltransferase), *tpi* (triosephosphate isomerase), *yqiL* (acetyl coenzyme A acetyletransferase) [50] were extracted from the genomic sequences of the seven representative *S. aureus* isolates and concatenated. Phylogenetic analysis on these sequences was performed using RaxML [42]. Sequence types for the isolates were obtained from the *S. aureus* MLST database based on the allelic profiles for each representative isolate.

### Structural modelling of SdrD

Amino acid sequences of seven representative *sdrD* variants were used in generating a 3D model for their SdrD structure. 3D structures were generated using the Swiss model server [51] based on the resolved crystal structure of the *S. aureus* SdrD N2-N3-B1 subdomains [13]. Further editing and visualization of structures were performed using PyMOL software [52] (Schrodinger, Inc. USA).

### *sdrD* cloning and complementation of *S. aureus* ∆*sdrD*

The open reading frame encoding the entire *sdrD* gene was amplified from the genomic DNA of the seven representative *S. aureus* strains using primers listed in Table 4. The seven amplified PCR products were ligated into the Gram-positive shuttle plasmid pMG36e [53] with erythromycin as the selectable marker (kind gift from Dr. Diep Dzung, Norwegian University of life Science, Norway) creating six pMG36e-*sdrD* plasmids. The constructed plasmids pMG36e-*sdrD* and empty pMG36e were propagated in *E. coli* DH5, under selective pressure (LB supplemented with 400 μg/ml erythromycin).

To overcome the restriction barrier, the plasmids were transformed into and purified from *E. coli* DC10b [54]. The plasmids were electroporated (2.1 kV voltage, 600-Ω resistance, 10 μF capacitance) into *S. aureus* NCTC8325∆*sdrD* [8] and transformants were selected at 30 °C on TSA supplemented with 8 μg/ml erythromycin. To confirm successful transformation of the isogenic *S. aureus* NCTC8325–4∆*sdrD,* plasmids were isolated from the recipient strains and confirmed by direct sequencing. Expression of SdrD by the complemented mutant was confirmed by immunoblotting as previously described [8].

### Bacterial growth measurement

Complemented *S. aureus* mutants were grown overnight at 30 °C in TSB containing 8 μg/ml erythromycin. Next day, the bacteria were diluted 1:150 into fresh pre-warmed TSB with 8 μg/ml erythromycin. Thereafter, 275 μl of the diluted culture was transferred to wells of Nunclon delta surface 96 well plate (Thermo Scientific, US) in replicates of five. A control containing only the growth medium was included in order to adjust for background fluorescence. The plate was maintained at continuous orbital shaking in a Synergy H1 Reader (BioTek, US). Growth was assayed by measuring optical density at 600 nm at every 15 mins for 24 h.

### Mammalian cell line

HaCaT cells, an immortalized human keratinocyte cell line [55], were purchased from CLS Cell Lines Service Germany (item number: 300493). Cells were maintained in Dulbecco’s modified Eagle’s medium (DMEM) (Sigma Aldrich, Germany), supplemented with 10% (*v*/v) fetal bovine serum (FBS) (Invitrogen Life Technologies, USA), penicillin (100 units/ml), and streptomycin 100 μg/ml (Sigma Aldrich, Germany) and at 37 °C in humidified 5% CO_2._ They were routinely sub cultured at 70%–80% confluency.

### Bacterial adhesion to HaCaT cells

HaCaT cells were seeded into a 24 well plate at a concentration of 1.5 – 2 × 10^5^ cells/ml in DMEM supplemented with 10% FBS. *S. aureus* NCTC8325∆*sdrD* complemented with the pMG36e-*sdrD* variants were grown overnight at 30 °C and 230 rpm in TSB with 8 μg/ml erythromycin. Next day, the overnight cultures were diluted into fresh medium and grown to OD_600nm_ of 0.7–0.8. The bacterial cultures were pelleted and washed with PBS. Subsequently, the bacteria were resuspended in DMEM supplemented with 10% FBS and added to the HaCaT cells at multiplicity of infection (MOI) of 100, and incubated at 37 °C for 90 mins. Thereafter, the HaCaT cells were treated, washed and the number of adhered bacteria enumerated as described earlier [8].

### Statistical analysis

Statistical analysis was carried out on the pooled data using GraphPad prism 7 (GraphPad Software Inc. USA). Statistical significance was quantified by one-way anova test with Tukey’s multiple comparison test. Data with *P* < 0.05 were considered statistically significant.

## Additional files

Additional file 1: Multiple Sequence Alignment of nucleotide sequences of SdrD A region for 48 queried and 6 reference strains. Consensus residues within the residues are indicated as conserved (*) and semi-conserved (.). Deletion and insertions are indicated as (−). (PDF 3240 kb)

Additional file 2: Multiple Sequence Alignment of deduced amino acid sequence of *sdrD* gene of the representative sdrD variants. The different domains within the SdrD structure are indicated. The YSIRK/GS and signal cleavage peptide within the signal sequences are indicated in red boxes. The predicted EF motifs within the B repeat and the LPXTG motif is indicated in red boxes also. Alignment was performed with MAFFT and edited using Boxshade and Adobe Photoshop. Consensus residues within the residues are indicated as conserved (*) and semi-conserved (.). Deletion and insertions are indicated as (−). (PDF 10515 kb)

Additional file 3: Growth curve measurement and SdrD Expression **(A)**: Growth curve measurement for the *S. aureus* isogenic mutant NCTC8325–4*ΔsdrD* complemented with either different pMG36e-SdrD variants (variant) or pMG36e (empty vector). Growth was measured as absorbance at OD =600 nm over 24 h. **(B**). Immunoblot for the detection of SdrD expression by the NCTC8325–4 (WT) NCTC8325–4*ΔsdrD* (∆*sdrD),* NCTC8325–4*ΔsdrD* complemented with either different pMG36e-SdrD variants (indicated by the number) or pMG36e (Empty). The molecular weight (Kda) is also indicated (PNG 234 kb)

Additional file 4: **Table S1** Percentage amino acid identity using the amino acid sequences for the SdrD A domain for the seven representative *sdrD* variants. (PNG 274 kb)

## Abbreviations

Dsg1: Desmoglein 1
FnBP: Fibronectin binding protein
HGT: Horizontal gene transfer
MGE: Mobile genetic element
MLST: Multilocus sequence typing
MSCRAMMs: Microbial surface component recognising adhesive matrix molecules
Sdr: Serine-aspartate-repeat protein
