# Supplementary material for: Genetic variability in the sdrD gene in Staphylococcus aureus from healthy nasal carriers
Source: BMC Microbiol. 2018 Apr 16;18:34. doi: 10.1186/s12866-018-1179-7 (PMC5902956; doi:10.1186/s12866-018-1179-7)

Additional File 1.

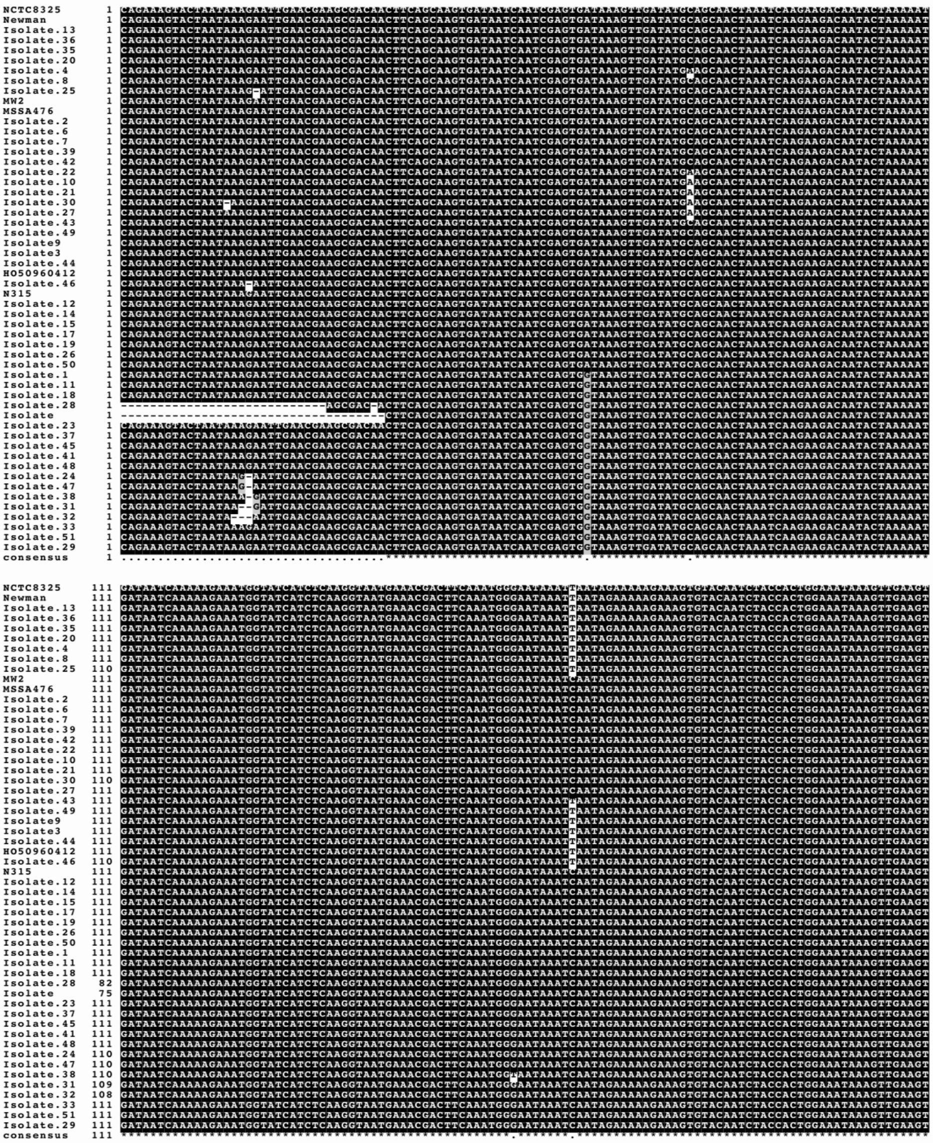

```
NCTC8325     221 TTCAACTGCCAAATCAGATGAGCAAGCTTCACCAAAATCTACGAATGAAGATTTAAACACTAAACAAACTATAAGTAATCAAGAAGCGTTACAACCTGATTTGCAAGAGA
Newman       221 TTCAACTGCCAAATCAGATGAGCAAGCTTCACCAAAATCTACGAATGAAGATTTAAACACTAAACAAACTATAAGTAATCAAGAAGCGTTACAACCTGATTTGCAAGAGA
Isolate.13   221 TTCAACTGCCAAATCAGATGAGCAAGCTTCACCAAAATCTACGAATGAAGATTTAAACACTAAACAAACTATAAGTAATCAAGAAGCGTTACAACCTGATTTGCAAGAGA
Isolate.36   221 TTCAACTGCCAAATCAGATGAGCAAGCTTCACCAAAATCTACGAATGAAGATTTAAACACTAAACAAACTATAAGTAATCAAGAAGCGTTACAACCTGATTTGCAAGAGA
Isolate.35   221 TTCAACTGCCAAATCAGATGAGCAAGCTTCACCAAAATCTACGAATGAAGATTTAAACACTAAACAAACTATAAGTAATCAAGAAGCGTTACAACCTGATTTGCAAGAGA
Isolate.20   221 TTCAACTGCCAAATCAGATGAGCAAGCTTCACCAAAATCTACGAATGAAGATTTAAACACTAAACAAACTATAAGTAATCAAGAAGCGTTACAACCTGATTTGCAAGAGA
Isolate.4    221 TTCAACTGCCAAATCAGATGAGCAAGCTTCACCAAAATCTACGAATGAAGATTTAAACACTAAACAAACTATAAGTAATCAAGAAGCGTTACAACCTGATTTGCAAGAGA
Isolate.8    221 TTCAACTGCCAAATCAGATGAGCAAGCTTCACCAAAATCTACGAATGAAGATTTAAACACTAAACAAACTATAAGTAATCAAGAAGCGTTACAACCTGATTTGCAAGAGA
Isolate.25   220 TTCAACTGCCAAATCAGATGAGCAAGCTTCACCAAAATCTACGAATGAAGATTTAAACACTAAACAAACTATAAGTAATCAAGAAGCGTTACAACCTGATTTGCAAGAGA
MW2          221 TTCAACTGCCAAATCAGATGAGCAAGCTTCACCAAAATCTACGAATGAAGATTTAAACACTAAACAAACTATAAGTAATCAAGAAGCGTTACAACCTGATTTGCAAGAGA
MSSA476      221 TTCAACTGCCAAATCAGATGAGCAAGCTTCACCAAAATCTACGAATGAAGATTTAAACACTAAACAAACTATAAGTAATCAAGAAGCGTTACAACCTGATTTGCAAGAGA
Isolate.2    221 TTCAACTGCCAAATCAGATGAGCAAGCTTCACCAAAATCTACGAATGAAGATTTAAACACTAAACAAACTATAAGTAATCAAGAAGCGTTACAACCTGATTTGCAAGAGA
Isolate.6    221 TTCAACTGCCAAATCAGATGAGCAAGCTTCACCAAAATCTACGAATGAAGATTTAAACACTAAACAAACTATAAGTAATCAAGAAGCGTTACAACCTGATTTGCAAGAGA
Isolate.7    221 TTCAACTGCCAAATCAGATGAGCAAGCTTCACCAAAATCTACGAATGAAGATTTAAACACTAAACAAACTATAAGTAATCAAGAAGCGTTACAACCTGATTTGCAAGAGA
Isolate.39   221 TTCAACTGCCAAATCAGATGAGCAAGCTTCACCAAAATCTACGAATGAAGATTTAAACACTAAACAAACTATAAGTAATCAAGAAGCGTTACAACCTGATTTGCAAGAGA
Isolate.42   221 TTCAACTGCCAAATCAGATGAGCAAGCTTCACCAAAATCTACGAATGAAGATTTAAACACTAAACAAACTATAAGTAATCAAGAAGCGTTACAACCTGATTTGCAAGAGA
Isolate.22   221 TTCAACTGCCAAATCAGATGAGCAAGCTTCACCAAAATCTACGAATGAAGATTTAAACACTAAACAAACAATAAGTAATCAAGAAGCGTTACAACCTGATTTGCAAGAGA
Isolate.10   221 TTCAACTGCCAAATCAGATGAGCAAGCTTCACCAAAATCTACGAATGAAGATTTAAACACTAAACAAACAATAAGTAATCAAGAAGCGTTACAACCTGATTTGCAAGAGA
Isolate.21   221 TTCAACTGCCAAATCAGATGAGCAAGCTTCACCAAAATCTACGAATGAAGATTTAAACACTAAACAAACAATAAGTAATCAAGAAGCGTTACAACCTGATTTGCAAGAGA
Isolate.30   220 TTCAACTGCCAAATCAGATGAGCAAGCTTCACCAAAATCTACGAATGAAGATTTAAACACTAAACAAACAATAAGTAATCAAGAAGCGTTACAACCTGATTTGCAAGAGA
Isolate.27   221 TTCAACTGCCAAATCAGATGAGCAAGCTTCACCAAAATCTACGAATGAAGATTTAAACACTAAACAAACAATAAGTAATCAAGAAGCGTTACAACCTGATTTGCAAGAGA
Isolate.43   221 TTCAACTGCCAAATCAGATGAGCAAGCTTCACCAAAATCTACGAATGAAGATTTAAACACTAAACAAACTATAAGTAATCAAGAAGCGTTACAACCTGATTTGCAAGAGA
Isolate.49   221 TTCAACTGCCAAATCAGATGAGCAAGCTTCACCAAAATCTACGAATGAAGATTTAAACACTAAACAAACTATAAGTAATCAAGAAGCGTTACAACCTGATTTGCAAGAGA
Isolate9     221 TTCAACTGCCAAATCAGATGAGCAAGCTTCACCAAAATCTACGAATGAAGATTTAAACACTAAACAAACTATAAGTAATCAAGAAGCGTTACAACCTGATTTGCAAGAGA
Isolate3     221 TTCAACTGCCAAATCAGATGAGCAAGCTTCACCAAAATCTACGAATGAAGATTTAAACACTAAACAAACTATAAGTAATCAAGAAGCGTTACAACCTGATTTGCAAGAGA
Isolate.44   221 TTCAACTGCCAAATCAGATGAGCAAGCTTCACCAAAATCTACGAATGAAGATTTAAACACTAAACAAACTATAAGTAATCAAGAAGCGTTACAACCTGATTTGCAAGAGA
HO50960412   221 TTCAACTGCCAAATCAGATGAGCAAGCTTCACCAAAATCTACGAATGAAGATTTAAACACTAAACAAACTATAAGTAATCAAGAAGCGTTACAACCTGATTTGCAAGAGA
Isolate.46   220 TTCAACTGCCAAATCAGATGAGCAAGCTTCACCAAAATCTACGAATGAAGATTTAAACACTAAACAAACTATAAGTAATCAAGAAGCGTTACAACCTGATTTGCAAGAGA
N315         221 TTCAACTGCCAAATCAGATGAGCAAGCTTCACCAAAATCTACGAATGAAGATTTAAACACTAAACAAACTATAAGTAATCAAGAAGGGTTACAACCTGATTTGCTAGAGA
Isolate.12   221 TTCAACTGCCAAATCAGATGAGCAAGCTTCACCAAAATCTACGAATGAAGATTTAAACACTAAACAAACTATAAGTAATCAAGAAGGGTTACAACCTGATTTGCTAGAGA
Isolate.14   221 TTCAACTGCCAAATCAGATGAGCAAGCTTCACCAAAATCTACGAATGAAGATTTAAACACTAAACAAACTATAAGTAATCAAGAAGGGTTACAACCTGATTTGCTAGAGA
Isolate.15   221 TTCAACTGCCAAATCAGATGAGCAAGCTTCACCAAAATCTACGAATGAAGATTTAAACACTAAACAAACTATAAGTAATCAAGAAGGGTTACAACCTGATTTGCTAGAGA
Isolate.17   221 TTCAACTGCCAAATCAGATGAGCAAGCTTCACCAAAATCTACGAATGAAGATTTAAACACTAAACAAACTATAAGTAATCAAGAAGGGTTACAACCTGATTTGCTAGAGA
Isolate.19   221 TTCAACTGCCAAATCAGATGAGCAAGCTTCACCAAAATCTACGAATGAAGATTTAAACACTAAACAAACTATAAGTAATCAAGAAGGGTTACAACCTGATTTGCTAGAGA
Isolate.26   221 TTCAACTGCCAAATCAGATGAGCAAGCTTCACCAAAATCTACGAATGAAGATTTAAACACTAAACAAACTATAAGTAATCAAGAAGGGTTACAACCTGATTTGCTAGAGA
Isolate.50   221 TTCAACTGCCAAATCAGATGAGCAAGCTTCACCAAAATCTACGAATGAAGATTTAAACACTAAACAAACTATAAGTAATCAAGAAGGGTTACAACCTGATTTGCTAGAGA
Isolate.1    221 TTCAACTGCCAAATCAGATGAGCAAGCTTCACCAAAATCTACGAATGAAGATTTAAACACTAAACAAACTATAAGTAATCAAGAAGCGTTACAACCTGATTTGCAAGAGA
Isolate.11   221 TTCAACTGCCAAATCAGATGAGCAAGCTTCACCAAAATCTACGAATGAAGATTTAAACACTAAACAAACTATAAGTAATCAAGAAGCGTTACAACCTGATTTGCAAGAGA
Isolate.18   221 TTCAACTGCCAAATCAGATGAGCAAGCTTCACCAAAATCTACGAATGAAGATTTAAACACTAAACAAACTATAAGTAATCAAGAAGCGTTACAACCTGATTTGCAAGAGA
Isolate.28   192 TTCAACTGCCAAATCAGATGAGCAAGCTTCACCAAAATCTACGAATGAAGATTTAAACACTAAACAAACTATAAGTAATCAAGAAGCGTTACAACCTGATTTGCAAGAGA
Isolate      185 TTCAACTGCCAAATCAGATGAGCAAGCTTCACCAAAATCTACGAATGAAGATTTAAACACTAAACAAACTATAAGTAATCAAGAAGCGTTACAACCTGATTTGCAAGAGA
Isolate.23   221 TTCAACTGCCAAATCAGATGAGCAAGCTTCACCAAAATCTACGAATGAAGATTTAAACACTAAACAAACTATAAGTAATCAAGAAGCGTTACAACCTGATTTGCAAGAGA
Isolate.37   221 TTCAACTGCCAAATCAGATGAGCAAGCTTCACCAAAATCTACGAATGAAGATTTAAACACTAAACAAACTATAAGTAATCAAGAAGCGTTACAACCTGATTTGCAAGAGA
Isolate.45   221 TTCAACTGCCAAATCAGATGAGCAAGCTTCACCAAAATCTACGAATGAAGATTTAAACACTAAACAAACTATAAGTAATCAAGAAGCGTTACAACCTGATTTGCAAGAGA
Isolate.41   221 TTCAACTGCCAAATCAGATGAGCAAGCTTCACCAAAATCTACGAATGAAGATTTAAACACTAAACAAACTATAAGTAATCAAGAAGCGTTACAACCTGATTTGCAAGAGA
Isolate.48   221 TTCAACTGCCAAATCAGATGAGCAAGCTTCACCAAAATCTACGAATGAAGATTTAAACACTAAACAAACTATAAGTAATCAAGAAGCGTTACAACCTGATTTGCAAGAGA
Isolate.24   220 TTCAACTGCCAAATCAGATGAGCAAGCTTCACCAAAATCTACGAATGAAGATTTAAACACTAAACAAACTATAAGTAATCAAGAAGCGTTACAACCTGATTTGCAAGAGA
Isolate.47   220 TTCAACTGCCAAATCAGATGAGCAAGCTTCACCAAAATCTACGAATGAAGATTTAAACACTAAACAAACTATAAGTAATCAAGAAGCGTTACAACCTGATTTGCAAGAGA
Isolate.38   220 TTCAACTGCCAAATCAGATGAGCAAGCTTCACCAAAATCTACGAATGAAGATTTAAACACTAAACAAACTATAAGTAATCAAGAAGCGTTACAACCTGATTTGCAAGAGA
Isolate.31   219 TTCAACTGCCAAATCAGATGAGCAAGCTTCACCAAAATCTACGAATGAAGATTTAAACACTAAACAAACTATAAGTAATCAAGAAGCGTTACAACCTGATTTGCAAGAGA
Isolate.32   218 TTCAACTGCCAAATCAGATGAGCAAGCTTCACCAAAATCTACGAATGAAGATTTAAACACTAAACAAACTATAAGTAATCAAGAAGCGTTACAACCTGATTTGCAAGAGA
Isolate.33   221 TTCAACTGCCAAATCAGATGAGCAAGCTTCACCAAAATCTACGAATGAAGATTTAAACACTAAACAAACTATAAGTAATCAAGAAGCGTTACAACCTGATTTGCAAGAGA
Isolate.51   221 TTCAACTGCCAAATCAGATGAGCAAGCTTCACCAAAATCTACGAATGAAGATTTAAACACTAAACAAACTATAAGTAATCAAGAAGCGTTACAACCTGATTTGCAAGAGA
Isolate.29   221 TTCAACTGCCAAATCAGATGAGCAAGCTTCACCAAAATCTACGAATGAAGATTTAAACACTAAACAAACTATAAGTAATCAAGAAGCGTTACAACCTGATTTGCAAGAGA
consensus    221 *********************************************************************.*****************.****************.*****

NCTC8325     331 ATAAATCAGTGGTAAATGTTCAACCAACTAATGAGGAAAACAAAAAGGTAGATGCCAAAACTGAATCAACTACATTAAATGTTAAAAGTGATGCTATCAAGAGTAATGAT
Newman       331 ATAAATCAGTGGTAAATGTTCAACCAACTAATGAGGAAAACAAAAAGGTAGATGCCAAAACTGAATCAACTACATTAAATGTTAAAAGTGATGCTATCAAGAGTAATGAT
Isolate.13   331 ATAAATCAGTGGTAAATGTTCAACCAACTAATGAGGAAAACAAAAAGGTAGATGCCAAAACTGAATCAACTACATTAAATGTTAAAAGTGATGCTATCAAGAGTAATGAT
Isolate.36   331 ATAAATCAGTGGTAAATGTTCAACCAACTAATGAGGAAAACAAAAAGGTAGATGCCAAAACTGAATCAACTACATTAAATGTTAAAAGTGATGCTATCAAGAGTAATGAT
Isolate.35   331 ATAAATCAGTGGTAAATGTTCAACCAACTAATGAGGAAAACAAAAAGGTAGATGCCAAAACTGAATCAACTACATTAAATGTTAAAAGTGATGCTATCAAGAGTAATGAT
Isolate.20   331 ATAAATCAGTGGTAAATGTTCAACCAACTAATGAGGAAAACAAAAAGGTAGATGCCAAAACTGAATCAACTACATTAAATGTTAAAAGTGATGCTATCAAGAGTAATGAT
Isolate.4    331 ATAAATCAGTGGTAAATGTTCAACCAACTAATGAGGAAAACAAAAAGGTAGATGCCAAAACTGAATCAACTACATTAAATGTTAAAAGTGATGCTATCAAGAGTAATGAT
Isolate.8    331 ATAAATCAGTGGTAAATGTTCAACCAACTAATGAGGAAAACAAAAAGGTAGATGCCAAAACTGAATCAACTACATTAAATGTTAAAAGTGATGCTATCAAGAGTAATGAT
Isolate.25   330 ATAAATCAGTGGTAAATGTTCAACCAACTAATGAGGAAAACAAAAAGGTAGATGCCAAAACTGAATCAACTACATTAAATGTTAAAAGTGATGCTATCAAGAGTAATGAT
MW2          331 ATAAATCAGTGGTAAATGCTCAACCAACTAATGAGGAAAACAAAAAGGTAGATGCCAAAACTGAATCAACTACATTAAATGTTAAAAGTGATGCTATCAAGAGTAATGCT
MSSA476      331 ATAAATCAGTGGTAAATGCTCAACCAACTAATGAGGAAAACAAAAAGGTAGATGCCAAAACTGAATCAACTACATTAAATGTTAAAAGTGATGCTATCAAGAGTAATGCT
Isolate.2    331 ATAAATCAGTGGTAAATGCTCAACCAACTAATGAGGAAAACAAAAAGGTAGATGCCAAAACTGAATCAACTACATTAAATGTTAAAAGTGATGCTATCAAGAGTAATGCT
Isolate.6    331 ATAAATCAGTGGTAAATGCTCAACCAACTAATGAGGAAAACAAAAAGGTAGATGCCAAAACTGAATCAACTACATTAAATGTTAAAAGTGATGCTATCAAGAGTAATGCT
Isolate.7    331 ATAAATCAGTGGTAAATGCTCAACCAACTAATGAGGAAAACAAAAAGGTAGATGCCAAAACTGAATCAACTACATTAAATGTTAAAAGTGATGCTATCAAGAGTAATGCT
Isolate.39   331 ATAAATCAGTGGTAAATGCTCAACCAACTAATGAGGAAAACAAAAAGGTAGATGCCAAAACTGAATCAACTACATTAAATGTTAAAAGTGATGCTATCAAGAGTAATGCT
Isolate.42   331 ATAAATCAGTGGTAAATGCTCAACCAACTAATGAGGAAAACAAAAAGGTAGATGCCAAAACTGAATCAACTACATTAAATGTTAAAAGTGATGCTATCAAGAGTAATGCT
Isolate.22   331 ATAAATCAGTGGTAAATGCTCAACCAACTAATGAGGAAAACAAAAAGGTAGATGCCAAAACTGAATCAACTACATTAAATGTTAAAAGTGATGCTATCAAGAGTAATGCT
Isolate.10   331 ATAAATCAGTGGTAAATGCTCAACCAACTAATGAGGAAAACAAAAAGGTAGATGCCAAAACTGAATCAACTACATTAAATGTTAAAAGTGATGCTATCAAGAGTAATGCT
Isolate.21   331 ATAAATCAGTGGTAAATGCTCAACCAACTAATGAGGAAAACAAAAAGGTAGATGCCAAAACTGAATCAACTACATTAAATGTTAAAAGTGATGCTATCAAGAGTAATGCT
Isolate.30   330 ATAAATCAGTGGTAAATGCTCAACCAACTAATGAGGAAAACAAAAAGGTAGATGCCAAAACTGAATCAACTACATTAAATGTTAAAAGTGATGCTATCAAGAGTAATGCT
Isolate.27   331 ATAAATCAGTGGTAAATGCTCAACCAACTAATGAGGAAAACAAAAAGGTAGATGCCAAAACTGAATCAACTACATTAAATGTTAAAAGTGATGCTATCAAGAGTAATGCT
Isolate.43   331 ATAAATCAGTGGTAAATGTTCAACCAACTAATGAGGAAAACAAAAAGGTAGATGCGAAAACTGAATCAACTACATTAAATGTTAAAAGTGATGCTATCAAGAGTAATGCT
Isolate.49   331 ATAAATCAGTGGTAAATGTTCAACCAACTAATGAGGAAAACAAAAAGGTAGATGCGAAAACTGAATCAACTACATTAAATGTTAAAAGTGATGCTATCAAGAGTAATGCT
Isolate9     331 ATAAATCAGTGGTAAATGTTCAACCAACTAATGAGGAAAACAAAAAGGTAGATGCCAAAACTGAATCAACTACATTAAATGTTAAAAGTGATGCTATCAAGAGTAATGAT
Isolate3     331 ATAAATCAGTGGTAAATGTTCAACCAACTAATGAGGAAAACAAAAAGGTAGATGCCAAAACTGAATCAACTACATTAAATGTTAAAAGTGATGCTATCAAGAGTAATGAT
Isolate.44   331 ATAAATCAGTGGTAAATGTTCAACCAACTAATGAGGAAAACAAAAAGGTAGATGCCAAAACTGAATCAACTACATTAAATGTTAAAAGTGATGCTATCAAGAGTAATGAT
HO50960412   331 ATAAATCAGTGGTAAATGTTCAACCAACTAATGAGGAAAACAAAAAGGTAGATGCCAAAACTGAATCAACTACATTAAATGTTAAAAGTGATGCTATCAAGAGTAATGAT
Isolate.46   330 ATAAATCAGTGGTAAATGTTCAACCAACTAATGAGGAAAACAAAAAGGTAGATGCCAAAACTGAATCAACTACATTAAATGTTAAAAGTGATGCTATCAAGAGTAATGAT
N315         331 ATAAATCAGTGGTAAATGTTCAACCAACTAATGAGGAAAACAAAAAGGTAGATGCGAAAACTGAATCAACTACATTAAATGTTAAAAGTGATGCTATCAAGAGTAATGCT
Isolate.12   331 ATAAATCAGTGGTAAATGTTCAACCAACTAATGAGGAAAACAAAAAGGTAGATGCGAAAACTGAATCAACTACATTAAATGTTAAAAGTGATGCTATCAAGAGTAATGCT
Isolate.14   331 ATAAATCAGTGGTAAATGTTCAACCAACTAATGAGGAAAACAAAAAGGTAGATGCGAAAACTGAATCAACTACATTAAATGTTAAAAGTGATGCTATCAAGAGTAATGCT
Isolate.15   331 ATAAATCAGTGGTAAATGTTCAACCAACTAATGAGGAAAACAAAAAGGTAGATGCGAAAACTGAATCAACTACATTAAATGTTAAAAGTGATGCTATCAAGAGTAATGCT
Isolate.17   331 ATAAATCAGTGGTAAATGTTCAACCAACTAATGAGGAAAACAAAAAGGTAGATGCGAAAACTGAATCAACTACATTAAATGTTAAAAGTGATGCTATCAAGAGTAATGCT
Isolate.19   331 ATAAATCAGTGGTAAATGTTCAACCAACTAATGAGGAAAACAAAAAGGTAGATGCGAAAACTGAATCAACTACATTAAATGTTAAAAGTGATGCTATCAAGAGTAATGCT
Isolate.26   331 ATAAATCAGTGGTAAATGTTCAACCAACTAATGAGGAAAACAAAAAGGTAGATGCGAAAACTGAATCAACTACATTAAATGTTAAAAGTGATGCTATCAAGAGTAATGCT
Isolate.50   331 ATAAATCAGTGGTAAATGTTCAACCAACTAATGAGGAAAACAAAAAGGTAGATGCGAAAACTGAATCAACTACATTAAATGTTAAAAGTGATGCTATCAAGAGTAATGCT
Isolate.1    331 ATAAATCAGTGGTAAATGCTCAACCAACTAATGAGGAAAACAAAAAGGTAGATGCCAAAACTGAATCAACTACATTAAATGTTAAAAGTGATGCTATCAAGAGTAATGCT
Isolate.11   331 ATAAATCAGTGGTAAATGCTCAACCAACTAATGAGGAAAACAAAAAGGTAGATGCCAAAACTGAATCAACTACATTAAATGTTAAAAGTGATGCTATCAAGAGTAATGCT
Isolate.18   331 ATAAATCAGTGGTAAATGCTCAACCAACTAATGAGGAAAACAAAAAGGTAGATGCCAAAACTGAATCAACTACATTAAATGTTAAAAGTGATGCTATCAAGAGTAATGCT
Isolate.28   302 ATAAATCAGTGGTAAATGCTCAACCAACTAATGAGGAAAACAAAAAGGTAGATGCCAAAACTGAATCAACTACATTAAATGTTAAAAGTGATGCTATCAAGAGTAATGCT
Isolate      295 ATAAATCAGTGGTAAATGCTCAACCAACTAATGAGGAAAACAAAAAGGTAGATGCCAAAACTGAATCAACTACATTAAATGTTAAAAGTGATGCTATCAAGAGTAATGCT
Isolate.23   331 ATAAATCAGTGGTAAATGCTCAACCAACTAATGAGGAAAACAAAAAGGTAGATGCCAAAACTGAATCAACTACATTAAATGTTAAAAGTGATGCTATCAAGAGTAATGCT
Isolate.37   331 ATAAATCAGTGGTAAATGCTCAACCAACTAATGAGGAAAACAAAAAGGTAGATGCCAAAACTGAATCAACTACATTAAATGTTAAAAGTGATGCTATCAAGAGTAATGCT
Isolate.45   331 ATAAATCAGTGGTAAATGCTCAACCAACTAATGAGGAAAACAAAAAGGTAGATGCCAAAACTGAATCAACTACATTAAATGTTAAAAGTGATGCTATCAAGAGTAATGCT
Isolate.41   331 ATAAATCAGTGGTAAATGCTCAACCAACTAATGAGGAAAACAAAAAGGTAGATGCCAAAACTGAATCAACTACATTAAATGTTAAAAGTGATGCTATCAAGAGTAATGCT
Isolate.48   331 ATAAATCAGTGGTAAATGCTCAACCAACTAATGAGGAAAACAAAAAGGTAGATGCCAAAACTGAATCAACTACATTAAATGTTAAAAGTGATGCTATCAAGAGTAATGCT
Isolate.24   330 ATAAATCAGTGGTAAATGCTCAACCAACTAATGAGGAAAACAAAAAGGTAGATGCCAAAACTGAATCAACTACATTAAATGTTAAAAGTGATGCTATCAAGAGTAATGCT
Isolate.47   330 ATAAATCAGTGGTAAATGCTCAACCAACTAATGAGGAAAACAAAAAGGTAGATGCCAAAACTGAATCAACTACATTAAATGTTAAAAGTGATGCTATCAAGAGTAATGCT
Isolate.38   330 ATAAATCAGTGGTAAATGCTCAACCAACTAATGAGGAAAACAAAAAGGTAGATGCCAAAACTGAATCAACTACATTAAATGTTAAAAGTGATGCTATCAAGAGTAATGCT
Isolate.31   329 ATAAATCAGTGGTAAATGCTCAACCAACTAATGAGGAAAACAAAAAGGTAGATGCCAAAACTGAATCAACTACATTAAATGTTAAAAGTGATGCTATCAAGAGTAATGCT
Isolate.32   328 ATAAATCAGTGGTAAATGCTCAACCAACTAATGAGGAAAACAAAAAGGTAGATGCCAAAACTGAATCAACTACATTAAATGTTAAAAGTGATGCTATCAAGAGTAATGCT
Isolate.33   331 ATAAATCAGTGGTAAATGCTCAACCAACTAATGAGGAAAACAAAAAGGTAGATGCCAAAACTGAATCAACTACATTAAATGTTAAAAGTGATGCTATCAAGAGTAATGCT
Isolate.51   331 ATAAATCAGTGGTAAATGCTCAACCAACTAATGAGGAAAACAAAAAGGTAGATGCCAAAACTGAATCAACTACATTAAATGTTAAAAGTGATGCTATCAAGAGTAATGCT
Isolate.29   331 ATAAATCAGTGGTAAATGCTCAACCAACTAATGAGGAAAACAAAAAGGTAGATGCCAAAACTGAATCAACTACATTAAATGTTAAAAGTGATGCTATCAAGAGTAATGCT
consensus    331 ******************.************************************.****************************************************.*
```

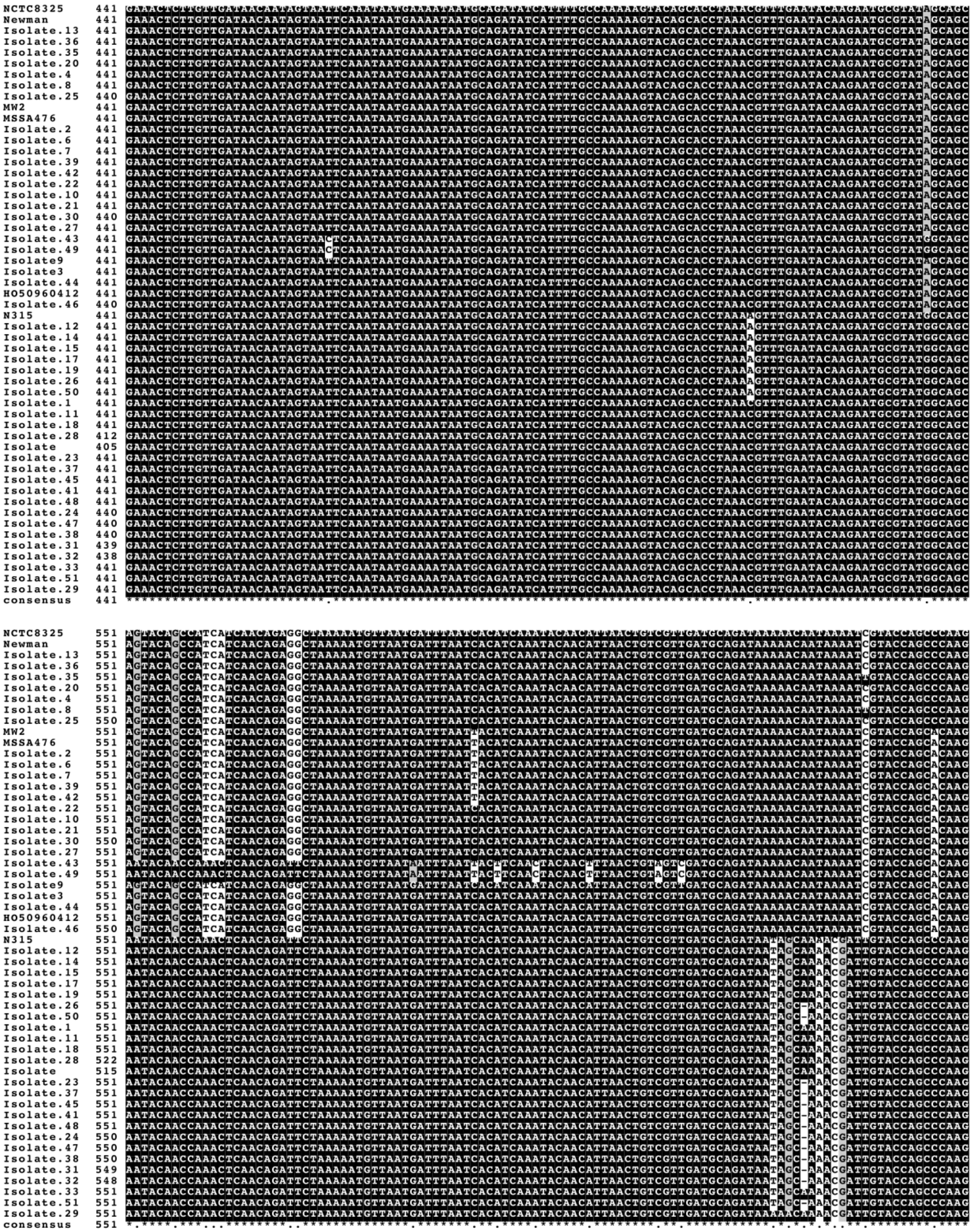

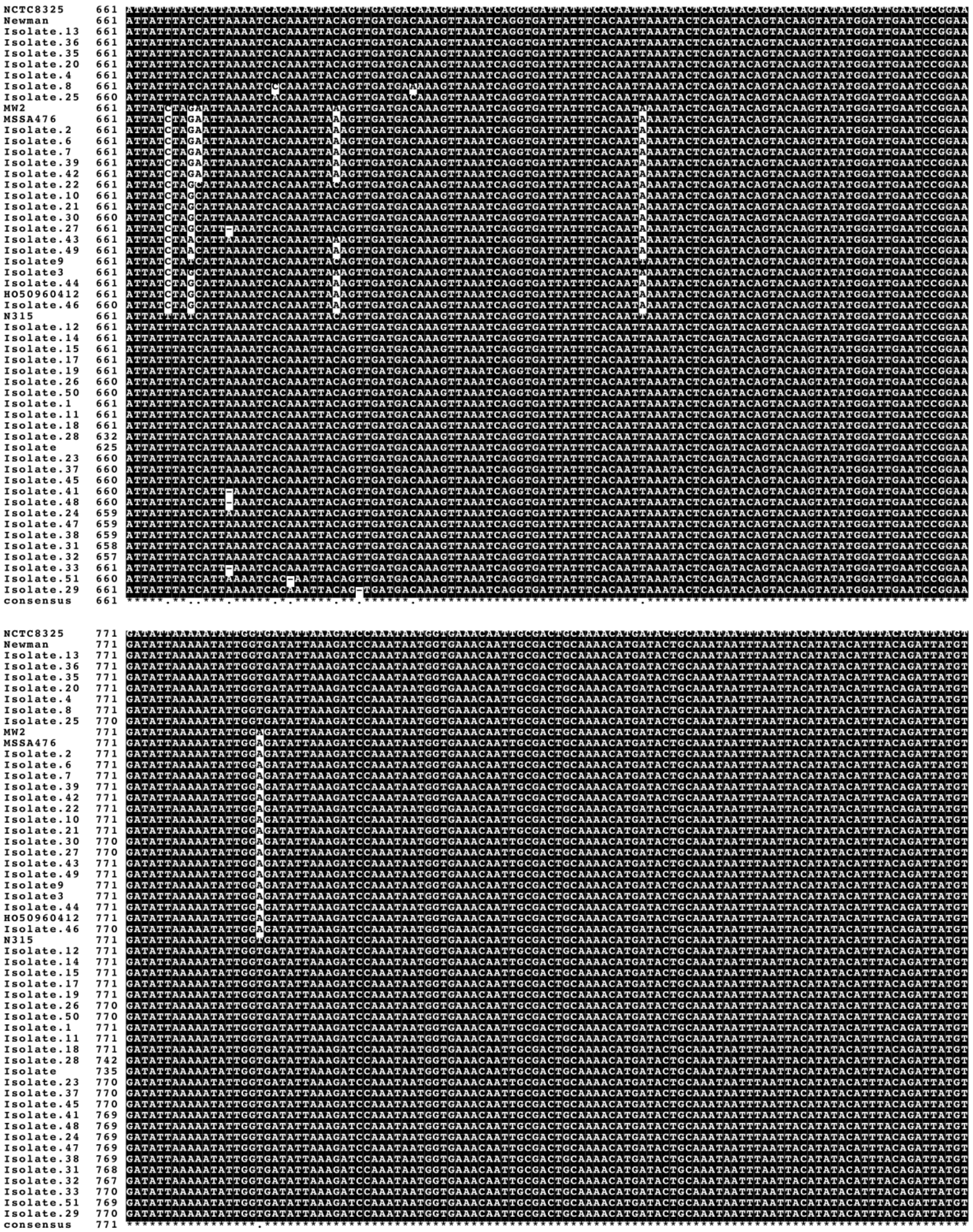

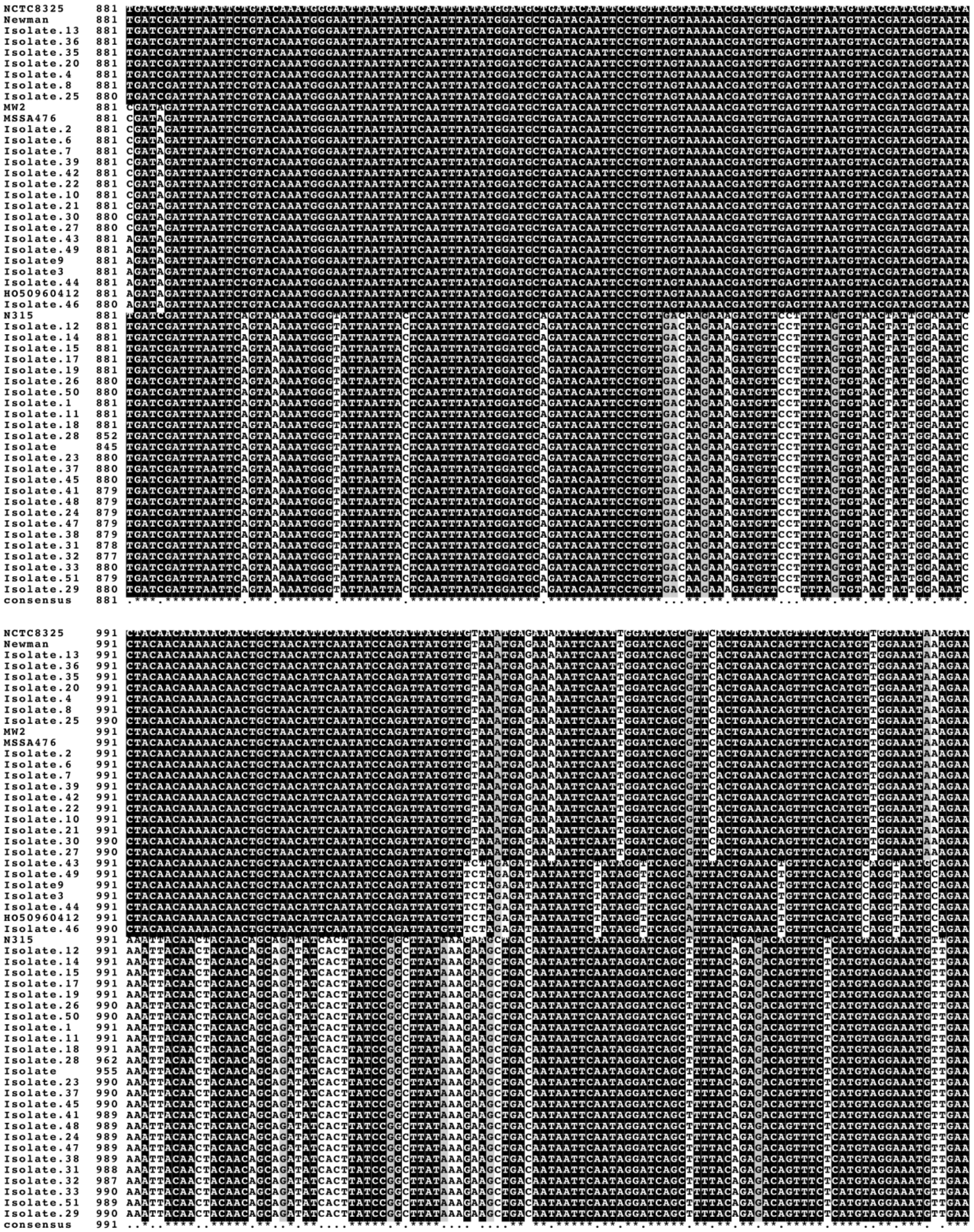

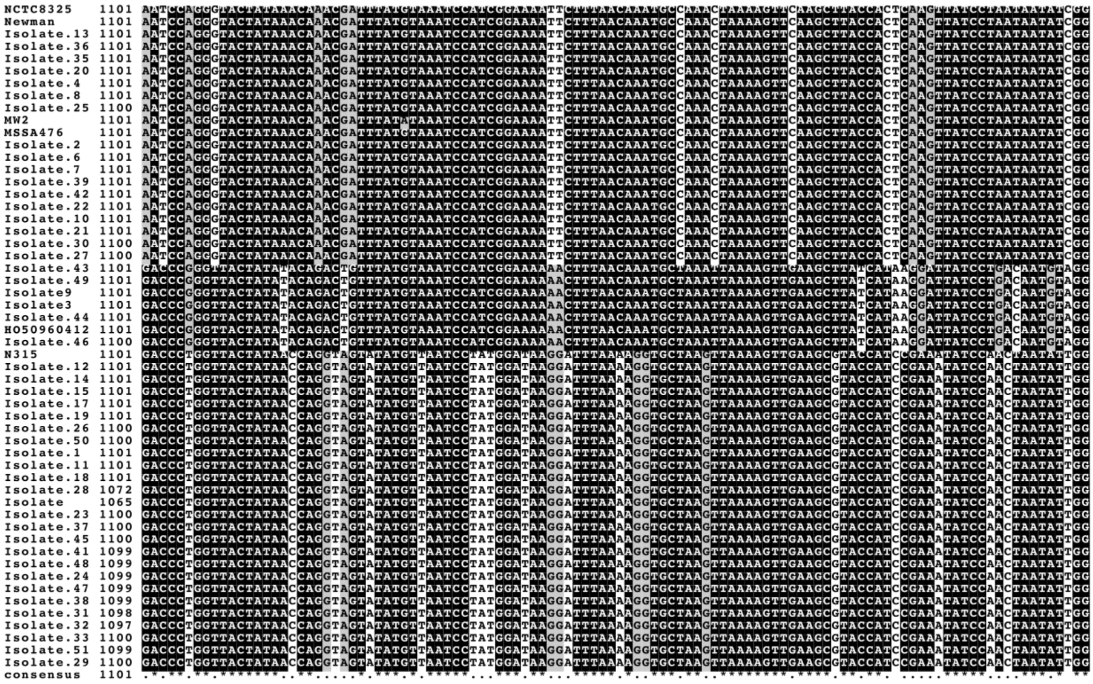

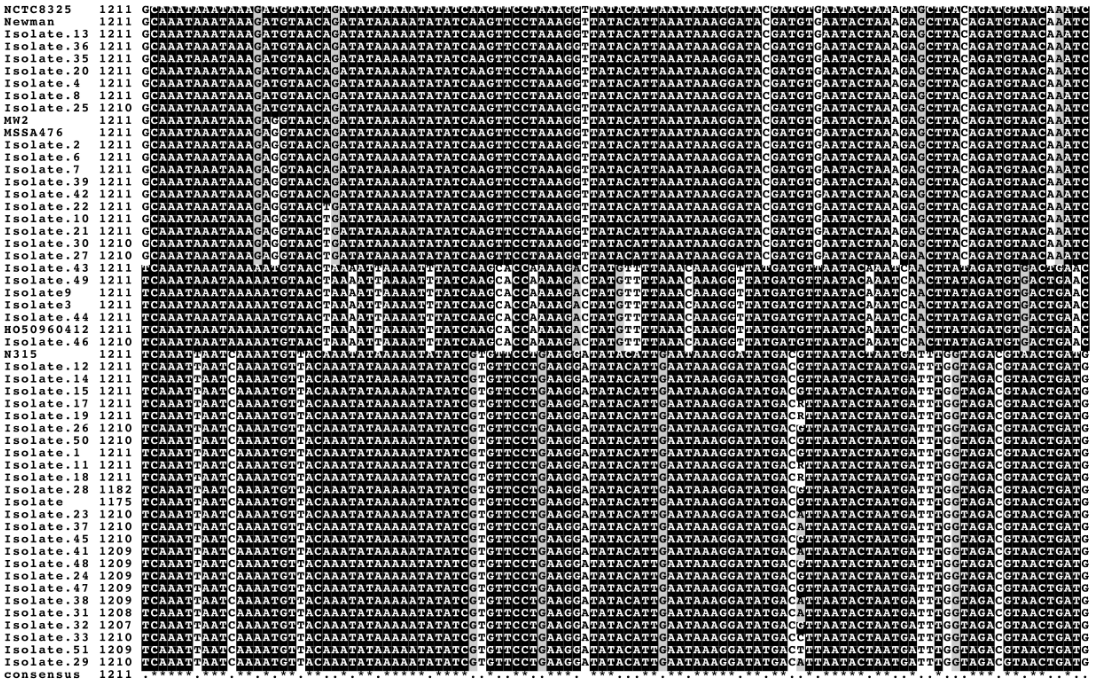

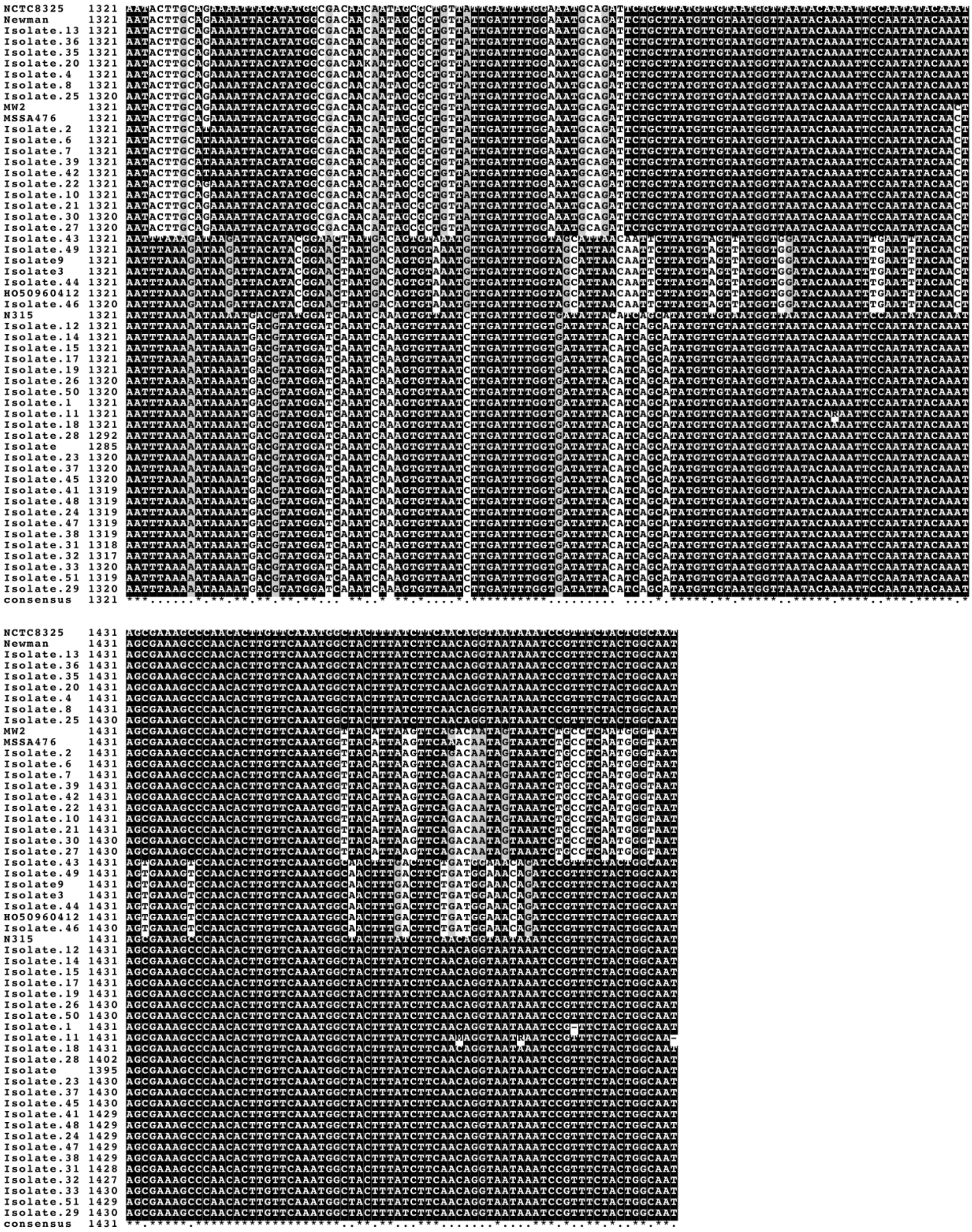

Supplement: Supplementary file 1 — Multiple Sequence Alignment of nucleotide sequences of SdrD A region for 48 queried and 6 reference strains. Consensus residues within the residues are indicated as conserved (*) and semi-conserved (.). Deletion and insertions are indicated as (−). (PDF 3240 kb) [file 12866_2018_1179_MOESM1_ESM.pdf]
