# Supplementary material for: Genetic variability in the sdrD gene in Staphylococcus aureus from healthy nasal carriers
Source: BMC Microbiol. 2018 Apr 16;18:34. doi: 10.1186/s12866-018-1179-7 (PMC5902956; doi:10.1186/s12866-018-1179-7)
Supplement: Supplementary file 2 — Multiple Sequence Alignment of deduced amino acid sequence of sdrD gene of the representative sdrD variants. The different domains within the SdrD structure are indicated. The YSIRK/GS and signal cleavage peptide within the signal sequences are indicated in red boxes. The predicted EF motifs within the B repeat and the LPXTG motif is indicated in red boxes also. Alignment was performed with MAFFT and edited using Boxshade and Adobe Photoshop. Consensus residues within the residues are indicated as conserved (*) and semi-conserved (.). Deletion and insertions are indicated as (−). (PDF 10515 kb) [file 12866_2018_1179_MOESM2_ESM.pdf]

Additional File 2

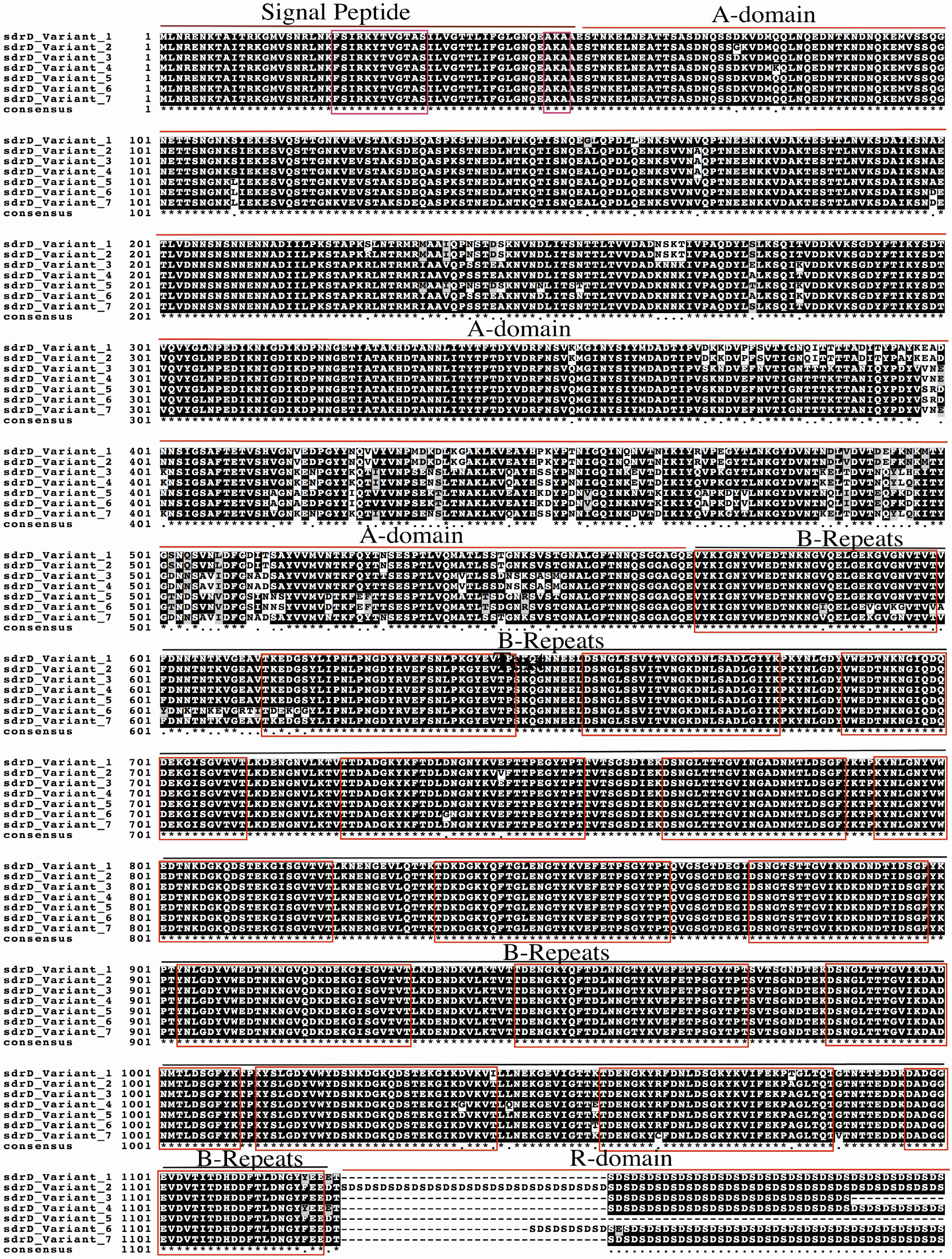

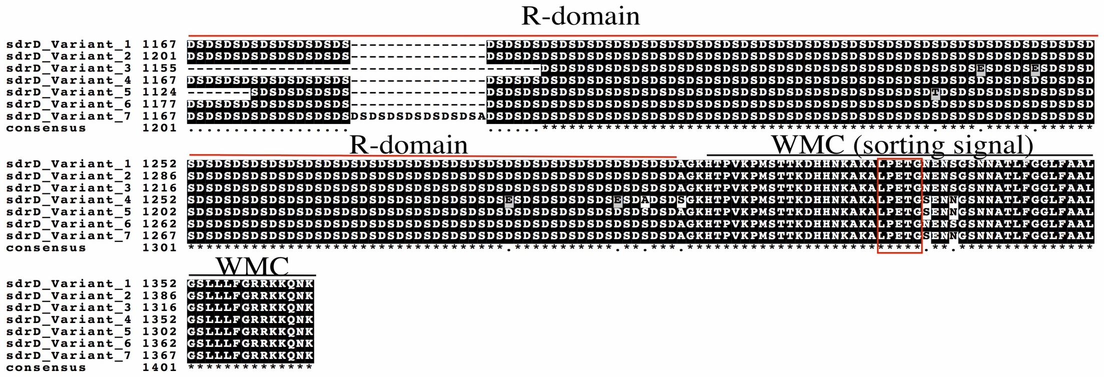
R-domain
R-domain
WMC (sorting signal)
WMC
