# Supplementary figures and images for: Genetic variability in the sdrD gene in Staphylococcus aureus from healthy nasal carriers
Source: BMC Microbiol. 2018 Apr 16;18:34. doi: 10.1186/s12866-018-1179-7 (PMC5902956; doi:10.1186/s12866-018-1179-7)

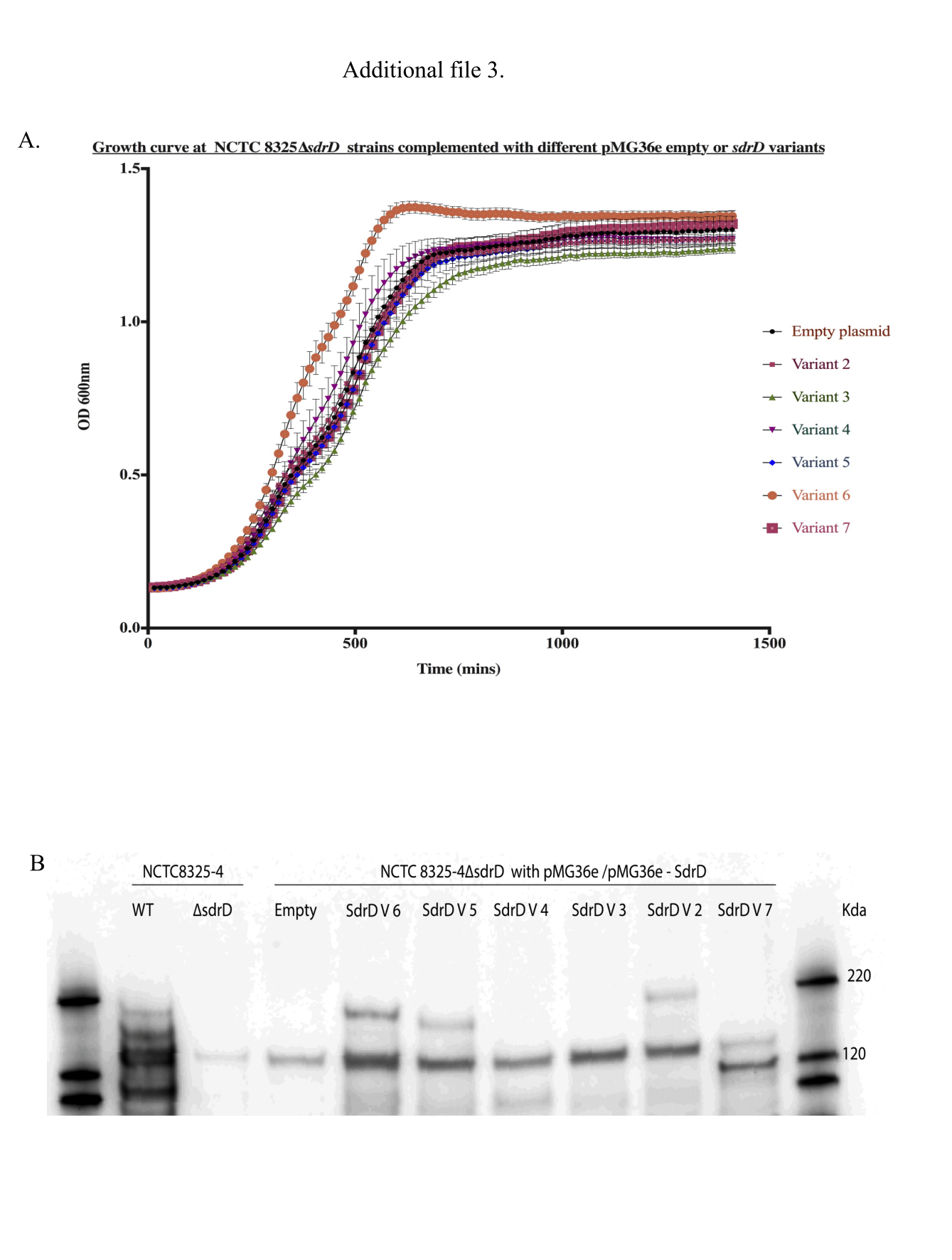

Supplement: Supplementary file 3 — Growth curve measurement and SdrD Expression (A): Growth curve measurement for the S. aureus isogenic mutant NCTC8325–4ΔsdrD complemented with either different pMG36e-SdrD variants (variant) or pMG36e (empty vector). Growth was measured as absorbance at OD =600 nm over 24 h. (B). Immunoblot for the detection of SdrD expression by the NCTC8325–4 (WT) NCTC8325–4ΔsdrD (∆sdrD), NCTC8325–4ΔsdrD complemented with either different pMG36e-SdrD variants (indicated by the number) or pMG36e (Empty). The molecular weight (Kda) is also indicated (PNG 234 kb) [file 12866_2018_1179_MOESM3_ESM.png]

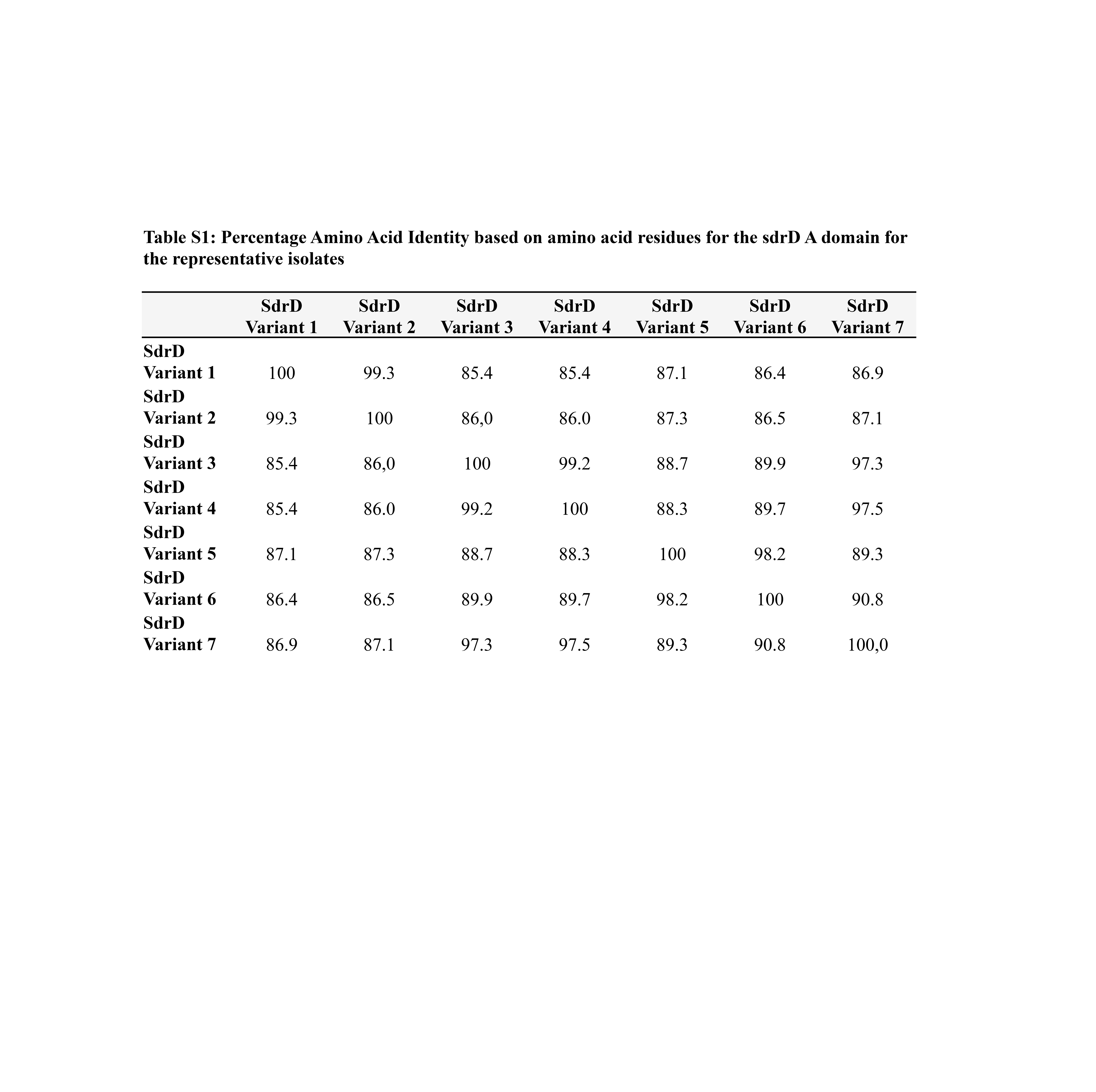

Supplement: Supplementary file 4 — Table S1 Percentage amino acid identity using the amino acid sequences for the SdrD A domain for the seven representative sdrD variants. (PNG 274 kb) [file 12866_2018_1179_MOESM4_ESM.png]
